# The relationship between Lp(a) and CVD outcomes: a systematic review

**DOI:** 10.1186/s12944-016-0258-8

**Published:** 2016-05-17

**Authors:** Carol A. Forbes, Ruben G. W. Quek, Sohan Deshpande, Gill Worthy, Robert Wolff, Lisa Stirk, Jos Kleijnen, Shravanthi R. Gandra, Stephen Djedjos, Nathan D. Wong

**Affiliations:** Kleijnen Systematic Reviews Ltd, Unit 6, Escrick Business Park, Riccall Road, Escrick, York, YO19 6FD UK; Amgen Inc, One Amgen Center Drive, Thousand Oaks, CA 91320-1799 USA; School for Public Health and Primary Care, Maastricht University, Maastricht, The Netherlands; University of California, Heart Disease Prevention Program, C240 Medical Sciences, University of California, Mail Code: 4079, Irvine, CA 92697 USA

**Keywords:** Atherosclerosis, Epidemiology, Lipids, Lipoprotein, Cardiovascular risk

## Abstract

Robust associations between lipoprotein(a) [Lp(a)] and CVD outcomes among general populations have been published in previous studies. However, associations in high risk primary prevention and secondary prevention populations are less well defined. In order to investigate this further, a systematic review was performed including prospective studies, which assessed the relationship between Lp(a) and CVD outcomes using multivariable analyses. Additional information was gathered on Lp(a) assays, multivariable modelling and population characteristics.

Literature searches from inception up to December 2015 retrieved 2850 records. From these 60 studies were included. Across 39 primary prevention studies in the general population (hazard ratios ranged from 1.16 to 2.97) and seven high risk primary prevention studies (hazard ratios ranged from 1.01 to 3.7), there was evidence of a statistically significant relationship between increased Lp(a) and an increased risk of future CVD. Results in 14 studies of secondary prevention populations were also suggestive of a modest statistically significant relationship (hazard ratios ranged from 0.75 to 3.7).

Therefore current evidence would suggest that increased Lp(a) levels are associated with modest increases in the risk of future CVD events in both general and higher risk populations. However, further studies are required to confirm these findings.

**Electronic supplementary material:**

The online version of this article (doi:10.1186/s12944-016-0258-8) contains supplementary material, which is available to authorized users.

## Background

Cardiovascular disease (CVD) is a leading cause of death and disability [1, 2]. Elevated levels of low-density lipoprotein cholesterol (LDL-C) are a major contributor to atherosclerosis leading to subsequent CVD events. Numerous clinical trials of lipid lowering drugs have found that reducing LDL-C levels substantially reduces the risk of CVD [3–5] suggesting a strong direct relationship between plasma LDL-C levels and CVD outcomes [6, 7]. Many people, however, still have residual CVD risk and suffer from CVD events despite significant LDL-C lowering. In addition to LDL-C, other risk factors are likely to influence residual cardiovascular risk. Among these, lipoprotein(a) [Lp(a)], has been proposed to be independently associated with CVD [8].

Lp(a) is an low density lipoprotein (LDL) particle which is attached to the polypeptide, apolipoprotein(a) [apo(a)] [9]. Apo(a) exists in multiple forms or ‘kringles’, which give rise to different Lp(a) isoforms. Apo(a) is also believed to be responsible for the anti-fibrinolytic properties of Lp(a) [9]. Further biomechanisms behind the Lp(a) and CVD relationship may also involve prothrombotic or proatherosclerotic processes, or a combination of the two [10].

Lp(a) may be measured using a variety of different assays. However, the reliability of many of the assays is questionable, due to their poor abilities at detecting the multiple molecular isoforms of Lp(a). Consequently, some assays (isoform dependent) that measure Lp(a) mass cannot distinguish between high and low molecular weight apo(a) isoforms, whilst others (isoform independent) can. However, to our knowledge, at present there appear to be no Lp(a) assays that are both isoform independent and suited for use clinical laboratories [11]. This problem has led to poor standardisation and comparability with respect to the Lp(a) values recorded by different assays, which in turn hampers comparisons between trials assessing the relationship between Lp(a) and CVD [12]. Despite this clinical trials have shown that Lp(a) is a risk factor in patients on long-term statin treatment [13, 14]. Evidence from the Atherothrombosis Intervention in Metabolic Syndrome with Low HDL/High Triglycerides: Impact on Global Health Outcomes (AIM-HIGH) trial suggests that Lp(a) is a predictor of CVD events in patients with normal LDL-C levels [15] and recent studies have suggested that elevated Lp(a) levels like elevated LDL-C, could be associated with premature CVD [8]. Extensive research exists to support an association between Lp(a) and CVD events with respect to the primary prevention of events in the general population [16, 17]. This relationship appears to be independent of LDL-C, other lipid levels such as high density lipoprotein (HDL) and the presence of other cardiovascular risk factors [18]. Some evidence from pooled analyses of prospective studies [19–21] suggests a potential association between Lp(a) and risk of CVD among high risk and secondary prevention population. The availability of new data from recently published clinical studies has prompted the need for a more contemporary systematic review. This review assesses the relationship between Lp(a) and CVD outcomes, with particular emphasis on high cardiovascular risk populations (high risk primary prevention and secondary prevention). The review also focuses on the best available evidence from studies that used multivariable analysis methods to control for the effect of confounding variables.

## Methods

To reduce the risks of bias and error, this review adhered to a pre-specified protocol and methods recommended by the Cochrane Collaboration [22], and the Centre for Reviews and Dissemination (York, United Kingdom) [23] which are widely regarded as ‘gold standard’ methodologies.

This review included prospective studies, which assessed the relationship between Lp(a) and CVD outcomes. These studies included randomised controlled trials (RCTs), cohort studies and nested case-control studies. Eligible populations were any adult (≥18 years) population regardless of baseline CVD risk, gender, age and ethnicity. Studies had to follow patients for at least 12 months. No restrictions were placed on the CVD outcome or the type of Lp(a) assay. However, studies were required to use a multivariable analytical approach, which assessed the effect of Lp(a) on CVD outcomes after adjusting for other confounding factors. At a minimum the analysis had to adjust for baseline age and gender; but this restriction was relaxed for studies in gender and age subgroups or in nested case-control studies where cases and controls were matched on age and gender. Studies were excluded from the review if they fail to clearly report effect sizes based on relevant multivariable analyses.

Extensive literature searches were performed using search strategies developed by an Information Specialist (full strategies are available in Additional file 1). A total of six electronic databases were searched from inception to 31 December 2015 including: MEDLINE, Embase, Medline In-Process & Daily Update, Cochrane Central Register of Controlled Trials (CENTRAL), Database of Abstracts of Reviews of Effects (DARE) and the Cochrane Database of Systematic Reviews (CDSR). Search strategies were refined and adapted according to the configuration and requirements of each database. The final strategies combined relevant search terms comprising indexed keywords (e.g. Medical Subject Headings, MeSH and EMTREE) and free text terms appearing in the title and/or abstract of database records. Search terms were identified through discussion between the review team, by scanning background literature and ‘key articles’ already known to the review team, and by browsing database thesauri. Literature searches were not limited by date, language or publication status. Supplementary searches were undertaken in two trials registers (National Institutes of Health [NIH] ClinicalTrials.gov and International Standard Randomised Controlled Trial Number [ISRCTN] Registry) and conference abstracts from four major cardiovascular disease conferences (European Atherosclerosis Society Congress; European Society of Cardiology Congress; American College of Cardiology Annual Scientific Session; and American Heart Association Annual Scientific Sessions, for years 2011-2015). The reference lists of included studies and systematic reviews were checked for further studies. Identified references were downloaded in Endnote X6 software (Thomson Reuters, New York) for further assessment and handling, and duplicate records were removed.

The study selection process was performed by two reviewers working independently. Data were extracted into a specifically developed spreadsheet in Excel 2010 (Microsoft Corporation, Redmond, Washington). One reviewer extracted the study data and a second reviewer independently reviewed the data against the original paper for completeness and accuracy. Data were extracted on the baseline population (e.g. race and previous CVD events), Lp(a) assay (e.g. isoform independence), CVD outcomes, statistical analysis methods (details of the type of multivariable model and the variables included) and effect sizes for the relationship between Lp(a) and CVD outcomes. The methodological quality (risk of bias) of each study was assessed using the criteria of the Quality in Prognostic Studies (QUIPS) tool [24]. The quality assessments were performed independently by two reviewers. Any discrepancies between reviewers during data extraction or quality assessments were resolved through consensus or consultation with a third reviewer.

Meta-analysis was not possible due to heterogeneity in the CVD outcomes, populations and statistical analysis methods. Studies have been summarized in a narrative synthesis accompanied by data tables. It was not possible to plot data on Forest plots due to the absence of necessary data. Effect sizes for Lp(a) are reported as odds ratios (ORs), hazard ratios (HRs) or adjusted Lp(a) levels with accompanying 95 % confidence intervals (CIs) or means/interquartile ranges (IQR). Studies are grouped according to CVD outcome, population and variables included in the multivariable model(s). The term “positive” association refers to an increase in Lp(a) or higher Lp(a) levels resulting in an increased risk of CVD outcomes. Similarly a “negative” association refers to an increase in Lp(a) or higher Lp(a) levels resulting in a decreased risk of CVD outcomes.

## Results

Literature searches of electronic databases and other sources including hand searching retrieved 3837 titles/abstracts through December 2015. After de-duplication, a total of 2850 titles/abstracts were screened, and 2189 papers were excluded as having no relevance to the review. Full papers of 312 potentially relevant references were selected for further examination. Of these, 197 papers were excluded after further examination for the following reasons: do not report relevant prognostic factors (20 papers), not relevant outcome (32 papers), not relevant study design (103 papers), and no clearly reported multivariable analysis (42 papers). A total of 60 studies (115 papers) met the criteria for inclusion in the review.

**Fig. 1:**
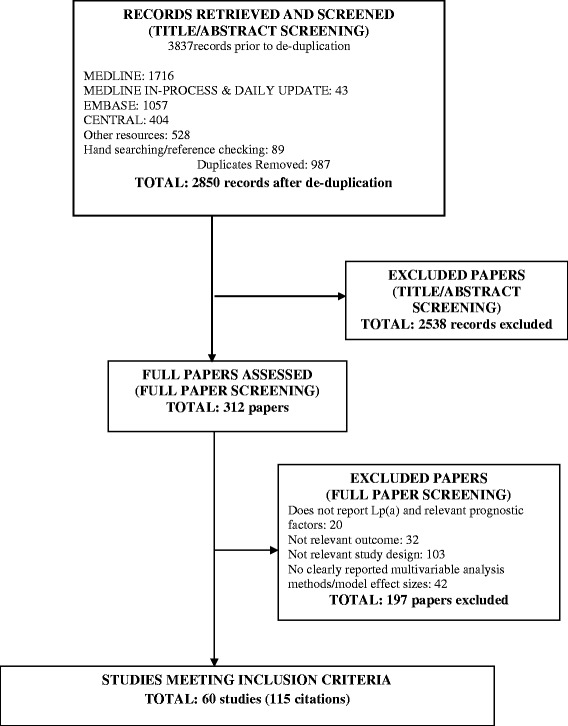
Preferred Reporting Items for Systematic Reviews and Meta-Analyses (PRISMA) flow diagram for the selection of studies

A summary of the identification and selection of studies for inclusion in this review is presented in Fig. 1, in accordance with the PRISMA [25].

The 60 studies included ten RCTs, 37 prospective cohort studies and 13 nested case control studies. A summary of the studies is reported in Table 1 and further information about individual studies is available in an Additional file 2: Table S1.

**Table 1:** Summary of characteristics across included studies (60 studies)

Study population	Study design (no. stds.)			Age (no. stds.)		Gender (no. stds.)			Ethnicity (no. stds.)			Risk of bias^a^(no. stds.)				Model type (no. stds.)						
	RCTs	Prospective cohort	Nested case control	< 65 years	≥ 65 years	Males only	Females only	Males and females	White only	Non-White only	Mixed or not reported/unclear	High	Moderate	Low	Not enough information	Cox proportional hazards	Logistic regression	Conditional &unconditional logistic	Discriminant analysis	Other	Conditional logistic regression	Includes LDL-C as model variable
Primary prevention General population (39 studies)	4	25	10	36	3	13	2	24	15	5	19	7	22	2	8	20	11	1	0	0	7	18
Primary prevention High risk population (7 studies)	1	6	0	7	0	1	0	6	1	2	4	4	1	1	1	4	1	0	0	1	0	1
Secondary prevention Previous CVD events (14 studies)	5	8	1	14	0	1	1	11	1	4	9	2	7	2	3	11	3	0	0	1	0	2
TOTAL (out of 60 studies)	10	39	11	57	3	15	3	41	17	11	32	13	30	5	11	35	15	1	0	2	7	21

no. number; std. studies

^**a**^Risk of bias according to Quality In Prognosis Studies (QUIPS) risk of bias assessment tool [24]

Overall, across all 60 studies, the level of bias was assessed as moderate. However 13 studies were assessed as having a high risk of bias and five studies as a low risk of bias; 11 studies failed to report sufficient detail so as to allow a full assessment of the risk of bias. Those studies that were of high risk of bias often had methodological issues within the QUIPS domains 2 and 3 concerning study attrition and prognostic factor [i.e. Lp(a)] measurement respectively. Reporting across studies was not always sufficiently detailed to allow a judgement to be made and 16 studies were reported as not having enough information to make a judgement for at least one of the six QUIPS criteria. Further details of the risk of bias assessments for the individual studies are available in a Additional file 3: Table S2.

### Primary prevention studies

The majority of the identified studies (39 studies) were carried out in participants from the general population, i.e. did not select patients based on their baseline history or risk of CVD events. Further details of the characteristics of the studies in the general population are reported in Table 2. These included four RCTs, 25 prospective cohort studies and 10 nested case-control studies. Thirteen studies [26–38] were conducted in males and two in females [39, 40]. Specific ethnic groups were used in some studies including populations from South Korea [41, 42], Native American Indians (from the USA) [43], Japan [44, 45] and Taiwan [46]. Follow-up in the studies tended to be longer than in the high risk and secondary prevention populations, with 20 out of 39 studies (52.3 %) having a follow-up of 5 to ≤ 10 years and 11 out of 39 studies; 28.2 %) following participants for over 10 yrs. The longest follow-up period was 20 years in the ARIC study [47]. The risk of bias across the 39 studies was assessed as low in two studies (5.4 %); moderate in 22 studies (84.0 %), high in seven studies (17.9 %) and there was insufficient information to make an assessment in eight (20.5 %) studies. Only 12 (30.8 %) studies used assays that were reported as isoform independent and five (12.8 %) used assays on fresh plasma samples. The majority of studies used a Cox proportional hazards model (20 studies; 51.3 %); other models included logistic regression (11 studies; 28.2 %), conditional logistic regression (seven studies; 17.9 %), and both conditional and unconditional logistic regression (one study; 2.6 %).

**Table 2:** Summary of primary prevention studies in the general population (39 studies)

Item	Category	Number of studies (%)
Study design	RCT	4/39 (10.3 %)
	Prospective cohort study	25/39 (64.1 %)
	Nested case-control study	10/39 (25.6 %)
Follow-up	1 yr to <2 yrs	1/39 (2.6 %)
	2 yrs to <5 yrs	4/39 (10.3 %)
	5 yrs to <10 yrs	20/39 (52.3 %)
	10 yrs+	11/39 (28.2 %)
	Not reported or unclear	3/39 (7.7 %)
Gender	Males only	13/39 (33.3 %)
	Females only	2/39 (5.1 %)
	Mixed males and females	24/39 (61.5 %)
Age	<65 yrs	36/39 (92.3 %)
	Elderly ≥ 65 yrs	3/39 (7.7 %)
Ethnicity	Mixed	3/39 (7.7 %)
	Korean	2/39 (5.1 %)
	Taiwanese	1/39 (2.6 %)
	Japanese	1/39 (2.6 %)
	Native American Indian	1/39 (2.6 %)
	White (majority populations)	15/39 (38.5 %)
	Not reported/unclear	16/39 (41.0 %)
Model method^a^	Cox proportional hazards	20/39 (51.3 %)
	Logistic regression	11/39 (28.2 %)
	Conditional and unconditional logistic regression	1/39 (2.6 %)
	Conditional logistic regression	7/39 (17.9 %)
	Discriminant analysis	0/39 (0 %)
Model variables	Includes LDL-C as model variable	19/39 (48.7 %)
	Does not include LDL-C or unclear/not reported	19/39 (48.7 %)
Lp(a) assay	Isoform dependent	1/39 (2.6 %)
	Isoform independent	12/39 (30.8 %)
	Isoform independent and dependent	1/39 (2.6 %)
	Isoform independence NR or unclear	25/39 (61.4 %)
Sample type	Fresh plasma samples	5/39 (12.8 %)
	Frozen plasma samples	25/39 (64.1 %)
	Mixture of frozen and fresh samples	2/39 (5.1 %)
	Not reported or unclear	7/39 (17.9 %)
Risk of bias	Low	2/39 (5.1 %)
	Moderate	22/39 (56.4 %)
	High	7/39 (17.9 %)
	Not enough information	8/39 (20.5 %)

*LDL-C* low density lipoprotein; Lp(a) lipoprotein (a); *NR* not reported; *RCT* randomised controlled trial; yrs years

^a^Note some studies report multiple types of models

Half of the studies included LDL-C (19 studies; 48.7 %) as a covariate in the multivariable model and all but four of these studies (Justification for the Use of Statins in Primary Prevention: An Intervention Trial Evaluating Rosuvastatin trial [JUPITER] [14], PRospective du l’Infarctus MyocardE; prospective epidemiological study of myocardial infarction [PRIME] [34], Invecchiare in Chianti [InCHIANTI] Study [48] and Atherosclerosis Risk in Communities [ARIC] [49]) reported significant positive associations between Lp(a) and CVD. The JUPITER study [14] and PRIME [34] each reported two negative associations between Lp(a) and CVD events, but in both cases these were for subgroup analyses and the results were not statistically significant. In the case of the JUPITER study [14] the authors concluded that among white participants treated with potent statin therapy, Lp(a) concentrations (at baseline and on-statin) were a significant determinant of residual risk with respect to CVD events; and in the PRIME study [34] that increased baseline Lp(a) levels (considered as the Lp(a) cholesterol content) were significantly associated with the risk for MI and angina pectoris, especially in men with high LDL-C. In the ARIC study [49] a significant negative association with Lp(a) mass was reported for a subgroup analysis of ischemic strokes in white females, when comparing >6.6 to ≤ 14.6 mg/dL Lp(a) [Quintile 4] versus 0.1 to ≤ 1.6/dL Lp(a) [Quintile 1]. However, this result was based on a small number of events suggesting that it may not be robust and was in contrast to the overall trend of the other primary and subgroup analyses, which suggested that overall a positive association existed between Lp(a) mass and CVD events. Overall, the authors concluded that Lp(a) mass was positively associated with CVD events, but that it appeared stronger in blacks compared to whites. The InCHIANTI study [48] did not report any statistically significant associations after following patients longitudinally for six years, but did find evidence of a link between prevalent peripheral arterial disease (PAD) and Lp(a). The authors concluded that Lp(a) concentration was an independent predictor of PAD in the cross-sectional evaluation, but that further larger, longer duration, prospective studies are needed to establish a longitudinal association.

Among the remaining half of the studies which did not include LDL-C as a covariate in their multivariable models, a further four studies (Physician’s Health Study [PHS] [33, 50], Italian Longitudinal Study on Aging [ILSA] [51] Uppsala Longitudinal Study of Adult Men [USLM] [26] and Women’s Health Initiative Observational Study/Hormones and Biomarkers Predicting Stroke Studies[WHI-OS/HaBPS] [39]) reported some negative associations between Lp(a) mass and CVD events. Negative statistically insignificant associations in the USLM study [26] were reported for the relationship between Lp(a) mass and intracerebral haemorrhage, but the authors concluded that high serum Lp(a) level independently predicted fatal and non-fatal stroke/transient ischemic attack (TIA) in a population of middle-aged men followed for 32 years. In the ILSA study [51] no overall statistically significant association was found between high Lp(a) levels and the risk of all-cause mortality, cumulative fatal–nonfatal stroke, and cumulative fatal–nonfatal coronary artery disease (CAD) events. However, the authors reported that high Lp(a) levels were an independent and significant predictor of non-fatal CAD events after 6.3 years in an elderly (65 to 84 years) population [51]. No association was also reported for Lp(a) concentration and ischemic stroke in the WHI-OS/HaBPS [39] study, where the authors concluded that they found no significant relationship between Lp(a) and ischemic stroke in postmenopausal women. However, the methods used to measure Lp(a) were not well described in this study and so their reliability was unclear. The PHS study [33] also found no evidence that Lp(a) levels were a significant predictor of PAD in men.

### High risk primary prevention studies

Seven studies assessed the relationship between Lp(a) and CVD outcomes in populations at high risk of CVD events, but who had not as yet experienced a CVD event (Agewall 2002; [52] Choices for Healthy Outcomes in Caring for End Stage Renal Disease study [CHOICE]; [53] Cleveland Clinic Hemodialysis Cohort; [54] Diamant Alpin Collaborative Dialysis Cohort; [55] Japan Diabetes Complications Study [JDCS]; [56] Koda 1999; [57] and Zimmermann 1999 [58]). Follow-up in the studies ranged from 2 years [14, 55, 57] to 7.8 years [56] and the sample size ranged from 118 [59] to 1494 [60] participants. These studies included patients with hypertension [52, 55], dialysis patients [55, 57, 58, 61, 62] and patients with diabetes [55–57, 63]. Five studies were in mixed gender populations [54–58, 61, 64], with one study in older (aged 56 to 77 yrs) males [52]. Two of the studies were in Japanese populations [56, 57]. A summary of the characteristics and effect sizes for these studies is shown in Table 3.

**Table 3:** Summary of primary prevention studies in high risk populations (7 studies)

Study Details	Analysis Methods	Summary of findings
Agewall 2002 [52] (*n* = 118) *Study design:* Prospective cohort study *Follow-up:* 3.0 ± 0.6 yrs *Population description:* Males 56 to 77 yrs with treated hypertension *Overall risk of bias* ^a^ *:* High risk *Funding:* NR	*Model:* Cox proportional hazards *Variables*: Age and other variables; LDL-C not included *CVD Outcomes:* Non-fatal MI or CD *Lp(a) assay:* Isoform independence - NR; NR if fresh or frozen samples	*Lp(a) comparison type:* Continuous Two effects sizes reported, each using a different model for the relationship between log per Lp(a) increase and non-fatal MI or CD: HR 2.84, 95 % CI: 1.06 to 7.63 (adjusted for age, BP, smoking, cholesterol, diabetes) HR 2.97, 95 % CI: 1.03 to 8.37 (adjusted for CD at entry) Both were statistically significant showing that Lp(a) is a significant and independent predictor for major coronary events
CHOICE [53] (*n* = 833) *Study design:* Prospective cohort study *Follow-up:* Median 27.4 mths *Population description:* Mixed gender adults 17 yrs + on dialysis *Overall risk of bias* ^a^ *:* Moderate risk *Funding:* Public/government	*Model:* Cox proportional hazards *Variables*: Age, gender and other variables; LDL-C not included *CVD Outcomes:* ASCVD *Lp(a) assay:* Isoform independent; frozen samples	*Lp(a) comparison type:* Categorical Ten effect sizes reported from five models of two categorical comparisons. Nine out of ten showed a statistically significant, positive association (same direction) for Lp(a) with respect to ASCVD. Maximum effect size reported was for Lp(a) ≥ 206 nmol/L (ref) vs. Lp(a) < 206 nmol/L (HR 1.89, 95 % CI: 1.3 to 2.75). One effect size was NS: Lp(a) ≥ 52.5 nmol/L (ref) vs. Lp(a) <52.5 nmol/L (HR 1.25, 95 % CI: 0.99 to 1.58) The authors concluded that ASCVD was significantly and independently associated with high Lp(a) (>123 nmol/L) and low molecular weight (LMW) apo(a) isoforms, though a stronger relationship was found for ASCVD and low molecular weight isoform size. This is in ESRD patients and there was a high transplantation rate (17.3 %) which may have biased the Lp(a) results
Cleveland Clinic Hemodialysis Cohort [54] (*n* = 129) *Study design:* Prospective cohort study *Follow-up:* 4 yrs *Population description:* Mixed gender adults ≥ 18 yrs on haemodialysis *Risk of bias assessment overall* ^a^ *:* Moderate risk *Funding:* Public/government	*Model:* Multiple regression (OR) and Cox proportional hazards (HR) *Variables*: Gender and other variables; includes LDL-C *CVD Outcomes:* Atherosclerotic events including stroke and MI *Lp(a) assay:* Isoform independence -NR/unclear; frozen samples	*Lp(a) comparison type:* Continuous Two effect sizes reported for Lp(a) with respect to atherosclerotic events: OR 1.02, 95 % CI: 1.01 to 1.04 (multiple regression) HR 1.603, 95 % CI: 1.08 to 2.38 (Cox proportional) A 1-mg/dL or 10-mg/dlL increment in baseline Lp(a) concentration was associated with a 1.02 or 1.26 increase, respectively, in the relative risk of sustaining an event (*p* = 0.001) Both results suggested that baseline Lp(a) is a significant and independent risk factor for clinical events
Diamant Alpin Collaborative Dialysis Cohort [55] (*n* = 279) *Study design:* Prospective cohort study *Follow-up:*2 yrs *Population description:* Mixed gender adults 22 to 92 yrs with and without type 2 diabetes *Risk of bias assessment overall* ^a^ *:* High risk *Funding:* Pharma	*Model:* Cox proportional hazards regression *Variables*: Age, gender and other variables; LDL-C not included *CVD Outcomes:* CVD events (MI, de novo angina pectoris or coronary revascularization, ischemic stroke, or PAD) and CV death (due to cardiac arrhythmia, MI, or HF) *Lp(a) assay:* NR/unclear; Immunoturbidimetric assay; Isoform dependence - NR/unclear; NR if fresh or frozen samples	*Lp(a) comparison type:* Categorical One effect size reported which showed a statistically significant positive association (same direction) for Lp(a) > 300 mg/L vs ≤ 300 with CVD events and CV deaths: HR 1.67, 95 % CI: 1.04 to 2.63 This result suggested that Lp(a) is an independent and significant predictor of CV events.
JDCS [56] (*n* = 1304) *Study design:* RCT *Follow-up:* Median 7.8 yrs *Population description:* Mixed gender Japanese adults 40 to 70 yrs with Type 2 diabetes *Risk of bias assessment overall* ^a^ *:* High risk *Funding:* Public/government	*Model:* Cox proportional hazards *Variables*: Age, gender and other variables; LDL-C not included *CVD Outcomes:* Stroke (ischemic, hemorrhagic or TIA), CHD *Lp(a) assay:* Isoform independence -NR/unclear; frozen samples	*Lp(a) comparison type:* Continuous One effect size reported which showed a statistically significant positive association (same direction) of Lp(a) (per 1 μmol/l increase) with an increased risk of stroke (ischemic, hemorrhagic or TIA): HR 1.16, 95 % CI: 1.03 to 1.31 Suggests that increasing Lp(a) is and independent and significant risk factor for stroke
Koda 1999 [57] (*n* = 390) *Study design:* Prospective cohort study *Follow-up:*2.3 yrs *Population description:* Mixed gender Japanese adults ≥ 18 yrs with or without type 2 diabetes receiving haemodialysis *Risk of bias assessment overall* ^a^ *:* High risk *Funding:* NR/unclear	*Model:* Multiple logistic regression model *Variables*: Age, gender, albumin, Lp(a), diabetic state; LDL-C not included *CVD Outcomes:* Death and CV death *Lp(a) assay:* NR/unclear; Immunoturbidimetric assay; Isoform dependence - NR/unclear; NR if fresh or frozen samples	*Lp(a) comparison type:* Categorical One effect size reported for Lp(a) showed a statistically significant positive association (same direction) with respect to CV death, comparing High Lp(a) [≥ 30 mg/dL] vs Low Lp(a) [< 30 mg/dL]: OR 3.93, 95 % CI: NR. This association was statistically significant. One effect size reported for Lp(a) with respect to overall death, comparing High Lp(a) [≥ 30 mg/dL] vs Low Lp(a) [< 30 mg/dL]: OR 1.97 95 % CI: NR. This association was not statistically significant. Suggests that high Lp(a) [≥ 30 mg/dL] is an independent and significant risk factor for atherosclerotic CV death, but not overall death.
Zimmermann 1999 [58] (*n* = 440) *Study design:* Prospective cohort study *Follow-up:* 12 and 24mths *Population description:* White mixed gender adults 20 to 88 yrs on chronic haemodialysis *Risk of bias assessment overall* ^a^ *:* Low risk *Funding:* Public/government	*Model:* Cox proportional hazards *Variables*: Age, gender and other variables; LDL-C not included *CVD Outcomes:* All deaths, stroke, HF, MI *Lp(a) assay:* Isoform independence - NR*;* fresh samples	*Lp(a) comparison type:* Categorical Lp(a) was significantly associated with risk of all-cause and cardiovascular mortality in univariate Cox regression analysis, but was not significant in the multivariable Cox regression analysis. This study is in haemodialysis patients, in such patients Lp(a) reacts as an acute phase protein in combination with other factors such as fibrinogen, HDL-C and Apo A-I, changing the atherogenic risk profile. When Lp(a) is added to the multivariable model with these other variables Lp(a) no longer remains significant as an independent factor.

^a^Risk of bias according to Quality In Prognosis Studies (QUIPS) risk of bias assessment tool [24]

ASCVD atherosclerotic cardiovascular disease; CD coronary death; CHD coronary heart disease; CHOICE Choices for Healthy Outcomes in Caring for ESRD; CI confidence interval; CVD cardiovascular events; ESRD end stage renal disease; HF heart failure; HR hazard ratio; JDCS Japan Diabetes Complications Study; LDL-C low density lipoprotein cholesterol; OR odds ratio; MI myocardial infarction; mth months; NR not reported; NS not statistically significant; RCT randomised controlled trial; TIA transient ischemic attack; yrs years

The overall quality of the six prospective cohort studies [53–55, 57, 58, 64] and one RCT [65] was mixed, with the risk of bias assessed as high in four studies [52, 55–57], moderate in two studies [54, 61] and low in one study [58].

CVD outcomes assessed in the studies were individual and composite outcomes including coronary heart disease (CHD) [56], non-fatal myocardial infarction (MI) [52, 55], cardiovascular death [52, 55, 57], atherosclerotic events [53, 54], non-haemorrhagic stroke [54, 65], heart failure (HF) [58], TIA [56] and all deaths [57, 58]. Six studies used Cox proportional hazards models [54–56, 58, 61, 64], with one study reporting results from stepwise multiple logistic and Cox regression analyses [54]. and one reporting a multiple logistic regression model [57]. Age was considered as a variable in all but one study [54], and similarly gender was considered in all but one study [52]. All of the studies included additional variables. These varied in type and number across the studies and studies reported different results for models adjusted with different groups of variables. However, only one study included LDL-C in their model [54]. This study (Cleveland Clinic Hemodialysis Cohort) [54] found that baseline Lp(a) concentration was a significant independent risk factor (*p* = 0.001) for clinical events attributed to atherosclerotic cardiovascular disease in patients receiving chronic haemodialysis treatment of end-stage renal disease. The only study to report the use of an isoform independent Lp(a) assay (CHOICE [53]) similarly reported that high Lp(a) levels (≥52.5 nmol/L) predicted a 30 % to 40 % increased risk of elevated atherosclerotic cardiovascular disease (ASCVD) in dialysis patients, but that the association of ASCVD with low molecular weight LMW isoforms (increased risk of 60 % to 90 %) was stronger than the association with high Lp(a) concentration.

In half of the studies Lp(a) levels were included as a continuous variable and the other half of the studies used categorical data. Of the eight studies, only one failed to find a significant relationship between Lp(a) and CVD outcomes (Zimmermann 1999 [58]). This study in haemodialysis patients reported that serum Lp(a) concentration was a significant predictor in univariate analyses, but that significance was lost when Lp(a) concentration was included in multivariable models for death and cardiovascular death [58]. This study was carried out in stable haemodialysis patients measuring outcomes at two years and the authors suggested that Lp(a) was only involved in an acute phase reaction. All of the remaining studies showed a positive association, i.e. that increased Lp(a) levels increased the risk of CVD events; hazard ratios (HRs) ranged from 1.16 to 2.97.

### Secondary prevention studies

Fourteen studies assessed the relationship between Lp(a) and CVD outcomes in patients with previous CVD events (Scandinavian Simvastatin Survival Study [4S Study]; [66] Atherothrombosis Intervention in Metabolic Syndrome With Low HDL/High Triglycerides: Impact on Global Health Outcome trial [AIM–HIGH]; [67] Ezhov 2014; [68] Global Evaluation of New Events and Restenosis After Stent Implantation [GENERATION] study; [69] Heart and Estrogen/progestin Replacement Study [HERS]; [70] Ikenaga 2011; [71] Park 2015; [72] Konishi 2013; [45] Kwon 2015; [73] Long-Term Intervention with Pravastatin in Ischaemic Disease [LIPID] study; [13] Rapamycin-Eluting Stent Evaluated At Rotterdam Cardiology Hospital study [RESEARCH]; [74] Rosengren 1990; [75] Treating to New Targets [TNT] study; [76] and Wehinger 1999 [77]). Follow-up in the studies ranged from 1 year [77] to 8.5 ± 3.5 years [68] and the sample size ranged from 115 [75] to 4444 [66] participants. These studies included patients with previous MI/CHD disease [63], [66, 70, 75, 76] CAD; [73] stent placement after symptomatic CAD [63, 72, 77], patients undergoing percutaneous coronary intervention (PCI) [45, 71, 78], history of CVD [13, 60, 67], patients after successful coronary artery bypass graft (CABG) [68] and patients hospitalised for stable angina, non-ST elevation acute coronary syndromes (NSTACS) or ST-segment elevation myocardial infarction (STEMI) [63, 69]. The majority of studies were in mixed gender populations with the exception of two studies in men [69, 75] and one in elderly postmenopausal women [70]. Two of the studies were in Japanese populations [45, 71] and two in Korean populations [72, 73]. A summary of the characteristics and effect sizes for these studies is shown in Table 4.

**Table 4:** Summary of studies in secondary prevention (14 studies)

Study Details	Analysis Methods	Summary of findings
4S Study [66] (*n* = 4444) *Study design:* RCT *Follow-up (median):* 5.4 yrs (range 4.9-6.3) *Population description:* Mixed gender aduts ≥ 18 yrs with history of CHD *Risk of bias assessment overall* ^a^ *:* Moderate *Funding:* Mixed (foundation/public)	*Model:* Logistic regresssion *Variables*: Age, gender and other variables; LDL-C not included *CVD Outcomes:* Death of any cause and MACE *Lp(a) assay:* Isoform independence – NR/unclear*;* frozen samples	*Lp(a) comparison type:* Categorical Comparisons included were: Lp(a) ≤ 38.25 units/l (ref) vs. 38.26-91 units/l, 91.1-289.75 units/l, ≥ 289.76 units/l No effect sizes reported, but all six logistic regression analyses reported a positive association, though this was only reported as statistically significant for three out of six comparisons (two simvastatin arm analyses and one placebo treatment arm). The authors concluded that Lp(a) independently predicts major coronary events as well as death in the secondary population.
AIM–HIGH [67] (*n* = 3414) *Study design:* RCT *Follow-up:* 3 yrs (at trial termination) *Population description:* Mixed gender adults ≥ 45 yrs with established CVD and dyslipidemia *Risk of bias assessment overall* ^a^ *:* Low *Funding:* Mixed (induxstry/public)	*Model:* Cox proportional hazards *Variables*: Age, gender and other variables; LDL-C not included *CVD Outcomes:* Ischemic stroke or TIA *Lp(a) assay:* Isoform independence - NR; NR if fresh or frozen samples	*Lp(a) comparison type:* Categorical Four effect sizes reported, including two for ischemic stroke and two for ischemic stroke or TIA. Comparisons were between lowest tertile (reference) Lp(a) and moderate tertile or highest tertile (Lp(a) levels not defined). All showed a statistically significant positive association (same direction). The maximum effect size reported was HR 2.8, 95 % CI: 1.25 to 6.26 (lowest tertile vs. highest tertile) and the lowest HR 2.3, 95 % CI: 1.19 to 4.42 (lowest tertile vs. highest tertile) Results show an independent and significant association between ischemic stroke and elevated baseline Lp(a) [middle/highest tertile]
Ezhov 2014 [68] (*n* = 356) *Study design:* Prospective cohort study *Follow-up:* 8.5 ± 3.5 yrs (range 0.9 -15.0 yrs) *Population description:* Mixed gender adults ≥ 18 yrs with stable CHD after sucessful CABG *Risk of bias assessment overall* ^a^ *:* Low *Funding:* NR	*Model:* Cox proportional hazards *Variables*: Age, gender and other variables; LDL-C not included *CVD Outcomes:* First cardiovascular event (non-fatal MI, cardiovascular death, coronary revascularization, or hospitalization for recurrent angina) *Lp(a) assay:* Isoform independent*;* fresh samples	*Lp(a) comparison type:* Categorical Two effect sizes reported, both were statistically significant showing a positive association (same direction) between Lp(a) and non-fatal MI or CD (< 30 mg/dl (reference) vs. ≥ 30 mg/dl: HR 2.98, 95 % CI: 1.76 to 5.03) and first ever major CVD event (< 30 mg/dl (ref) vs. ≥ 30 mg/dl: HR 3.47, 95 % CI: 2.48 to 4.85) These results show that Lp(a) concentration is independently associated with three-fold increase in risk of major adverse cardiovascular events within 15 years after CABG.
GENERATION [69] (*n* = 483) *Study design:* Prospective cohort study *Follow-up:* 1.84 yrs *Population description:* Males adults ≥ 18 yrs admitted to hospital with the diagnosis of either stable angina, NSTACS or STEMI *Risk of bias assessment overall* ^a^ *:* Not enough information *Funding:* NR	*Model:* Cox proportional hazards *Variables*: Age, gender and other variables; LDL-C not included *CVD Outcomes:* Cardiac death; non-fatal MI; rehospitalisation for rest-unstable angina *Lp(a) assay:* Isoform independence – NR/unclear; frozen samples	*Lp(a) comparison type:* Categorical Four effect sizes reported for comparison of < 25 mg/dl (reference) vs. ≥ 25 mg/dl. Three were statistically significant showing a positive association (same direction): HR 3.31, 95 % CI: 1.33 to 8.22 (non-fatal MI); HR 2.09, 95 % CI: 1.16 to 4.12 (rehosptialisation for angina); HR 2.42, 95 % CI: 1.52 to 3.84 (CD, non-fatal MI, hospitalisation for unstable angina) One was NS: HR 1.27, 95 % CI: 0.48 to 3.34 (death) Authors concluded that high plasma levels of either CRP or Lp(a) may be associated with the incidence of late events after successful coronary stenting, but a more protracted latent period may be needed in order to manifest clinically the unfavorable influence of an elevated Lp(a) on atherosclerotic plaque instability. The authors also noted that there was not a statndardised analytic method for Lp(a) level determination.
HERS [70] (*n* = 2759) *Study design:* RCT *Follow-up:* 4.1 yrs *Population description:* White elderly ≥ 50 yrs postmenopausal females with CHD – placebo group from RCT *Risk of bias assessment overall* ^a^ *:* Not enough information *Funding:* Industry	*Model:* Cox proportional hazards *Variables*: Age, gender and other variables; includes LDL-C *CVD Outcomes:* Unstable angina; primary CHD events including non-fatal MI and CHD death; MI *Lp(a) assay:* Isoform independent*;* NR if fresh or frozen samples	*Lp(a) comparison type:* Categorical 15 effect sizes reported for 5 different sets of CVD events. Only 2/15 analyses were statistically significant showing a positive association (same direction): 1^st^ quartile (Lp(a) 0.0-7.0 mg/dl - reference) vs. 4^th^ quartile (Lp(a) 55.0-236 mg/dl):HR 1.54, 95 % CI: 1 to 2.4 (primary CHD events, e.g non-fatal MI) 1^st^ quartile (Lp(a) 0.0-7.0 mg/dl - reference) vs. 4^th^ quartile (Lp(a) 55.0-236 mg/dl):HR 1.61, 95 % CI: 1.1 to 2.3 (CABG/PTCA) Overall, the authors concluded that Lp(a) is an independent risk factor for recurrent CHD in postmenopausal women.
Ikenaga 2011 [71] (*n* = 410) *Study design:* Prospective cohort study *Follow-up:* 5 yrs *Population description:* Japanese mixed gender adults ≥ 18 yrs with PCI after MI: Lp(a) ≥ 40 mg/dl *Risk of bias assessment overall* ^a^ *:* Not enough information *Funding:* No financial support	*Model:* Cox proportional hazards *Variables*: Age, gender and other variables; LDL-C not included *CVD Outcomes:* MACE (cardiac death, MI and/or revascularisation for new lesions); revascularisation for new lesions *Lp(a) assay:* Isoform independence – NR/unclear; NR if fresh or frozen samples	*Lp(a) comparison type:* Categorical Two effect sizes reported and both statistically significant showing a positive association: ≤ 40 mg/dl (reference) vs. > 40 mg/dl: HR 1.64, 95 % CI: 1.31 to 2.06 (MACE) ≤ 40 mg/dl (reference) vs. > 40 mg/dl: HR 1.61, 95 % CI: 1.32 to 2.13 (revascularisation for new lesions) Results show that Lp(a) levels can significantly and independently predict the progression of non-culprit lesions after acute MI
Konishi 2013 [45] (*n* = 330) *Study design:* Prospective cohort study *Follow-up (median):* 4.7 yrs *Population description:* Mixed gender Japenese adults ≥ 18 yrs undergoing PCI with achieved lipid targets: Lp(a) ≥30 mg/dl *Risk of bias assessment overall* ^a^ *:* High *Funding:* Public	*Model:* Cox proportional hazards and multivariable analysis *Variables*: Age, gender and other variables; LDL-C not included *CVD Outcomes:* All-cause death and ACS *Lp(a) assay:* Isoform independence – NR/unclear; NR if fresh or frozen samples	*Lp(a) comparison type:* Categorical Two effect sizes reported and both statistically significant showing a positive association (same direction): ≤ 30 mg/dl (reference) vs. ≥30 mg/dl: HR 1.68, 95 % CI: 1.03 to 2.7 (Cox proportional hazards) ≤ 30 mg/dl (reference) vs. ≥30 mg/dl: HR 2.47, 95 % CI: 1.19 to 5.06 (multivariable analysis) Results showed that high Lp(a) [ ≥30 mg/dL] could independently predict major adverse events
Kwon 2015 [73] (*n* = 1494) *Study design:* Prospective cohort study *Follow-up (mean):* 4.4 (SD 2.6) yrs *Population description:* Mixed gender Korean adults ≥ 18 yrs with diabetes and a history of symptomatic CAD including IHD, stable/unstable angina, and MI *Risk of bias assessment overall* ^a^ *:* Moderate *Funding:* Public	*Model:* Cox proportional regression analysis *Variables*: Age, gender and other variables; LDL-C leven not included (hyperlipidemia defined as LDL-C of at least 130 mg/dL was included) *CVD Outcomes:* MACE *Lp(a) assay:* Isoform independent; NR if fresh or frozen samples	*Lp(a) comparison type:* Categorical Two effect sizes (adjusting for age, gender, hypertension, hyperlipidemia, smoking and extent of CAD) reported and both statistically significant showing a positive association (same direction) with risk of MACE: Tertile 1 (median 4.7 mg/dL; reference) vs. Tertile 2 (median 13.5 mg/dL): HR 1.54, 95 % CI: 0.68 to 3.50 Tertile 1 (median 4.7 mg/dL; reference) vs. Tertile 3 (median 38.8 mg/dL): HR 2.89, 95 % CI: 1.37 to 6.08 In addition, a survival probability plot according to Lp(a) tertile suggested that elevated Lp(a) level was associated with a worse prognosis (*p* = 0,008) after adjusting for age, gender, hypertension, hyper lipidemia, smoking and extent of CAD. Results suggested elevated Lp(a) is associated with worse outcomes (MACE) in type 2 diabetics patients with symptomatic CAD and has incremental prognostic value.
LIPID [13] (*n* = 3949) *Study design:* RCT *Follow-up:* 6 yrs and 8 yrs *Population description:* Mixed gender White adults ≥ 18 yrs with history of CVD (Lp(a) 13.9-44.1 mg/dl) *Risk of bias assessment overall* ^a^ *:* Moderate *Funding:* Industry	*Model:* Cox proportional hazards *Variables*: Age, gender and other variables; LDL-C not included *CVD Outcomes:* Total CHD events (non-fatal MI, CHD death, unstable angina, coronary revascularization) *Lp(a) assay:* Isoform independent*;* frozen samples	*Lp(a) comparison type:* Categorical 36 effect sizes were reported across 3 different comparisons and 12 different CVD outcomes. 11/39 effect sizes were statistically significant analyses (all positive association (same direction). Effect size ranges were: From ≤ 13.9 mg/dl (reference) vs. >73.7 mg/dl: HR 1.21, 95 % CI: 1.07 to 1.36) To ≤ 13.9 mg/dl (reference) vs. >73.7 mg/dl: HR 1.45, 95 % CI: 1.2 to 1.75 28/39 analyses were NS including 3 at 8 yr follow-up and 28 at 6 yr follow-up Overall, the authors concluded that baseline Lp(a) and increased Lp(a) concentrations after one year were independently associated with future cardiovascular disease and CHD events.
Park 2015 [72] (*n* = 161) *Study design:* Prospective cohort study (retrospective analysis of prospective registry data) *Follow-up (median):* 6 yrs (maximum: 8 yrs) *Population description:* Mixed gender adults ≥ 18 yrs undergoing PCI *Risk of bias assessment overall* ^a^ *:* Moderate *Funding:* NR	*Model:* Cox proportional hazards *Variables*: Age, gender and other variables; LDL-C included *CVD Outcomes:* MACE *Lp(a) assay:* Isoform independence – NR/unclear*;* NR if fresh or frozen samples	*Lp(a) comparison type:* Categorical Cox proportional hazards regression analysis adjusted for gender, age, diabetes mellitus, hypertension, hyperlipidemia, smoking, multivessel disease, minimal luminal diameter after PCI, reference vessel diameter after PCI, LDL-C, total lesion length, Lp(a) ≥ 50 mg/dL, showed that Lp(a) > 50 mg/dL (vs. Lp(a) ≤ 50 mg/dL) was significantly associated with the 3 yr adverse clinical outcomes including any MI, revascularization (target lesion revascularization (TLR) and target vessel revascularization (TVR)), TLR- MACEs, TVR-MACE, and All-MACEs. One significant effect size: OR 2.88, 95 % CI: 1.37 to 6.07. Authors concluded that high Lp(a) level ≥ 50 mg/dL in angina pectoris patients undergoing elective PCI with DES was significantly associated with binary restenosis and 3 yr adverse clinical outcomes in an Asian population.
RESEARCH [74] (*n* = 161) *Study design:* Prospective cohort study *Follow-up (median):* 6 yrs (maximum: 8 yrs) *Population description:* Mixed gender adults ≥ 18 yrs undergoing PCI *Risk of bias assessment overall* ^a^ *:* Moderate *Funding:* NR	*Model:* Cox proportional hazards *Variables*: Age, gender and other variables; LDL-C not included *CVD Outcomes:* MACE *Lp(a) assay:* Isoform independent*;* frozen samples	*Lp(a) comparison type:* Categorical Eight effect sizes reported. Five out of eight effect sizes were statistically significant and all were positive association (same direction). These ranged from: Lp(a) tertile 1 (cut point 0.27 mg/dl - reference) vs. Lp(a) tertile 3 (> 1.83 mg/dl): HR 1.9, 95 % CI: 1 to 3.5 To Lp(a) tertile 1 (cut point 0.27 mg/dl - reference) vs. Lp(a) tertile 2 (cutpoint 1.83 mg/dl: HR 3.7, 95 % CI: 1.4 to 10.1 Three out of eight analyses were NS The results showed that high levels of Lp(a) were independently associated with a higher 1-year risk of MACE,
Rosengren 1990 [75] (*n* = 155) *Study design:* Nested case-control study *Follow-up:* 6 yrs *Population description:* Males ≥ 50 yrs with MI or CHD death *Risk of bias assessment overall* ^a^ *:* Moderate *Funding:* Mixed (foundation/ public)	*Model:* Logistic regression *Variables*: Age, gender and other variables; LDL-C not included *CVD Outcomes:* CHD deaths and non-fatal MI *Lp(a) assay:* Isoform independence – NR/unclear; frozen samples	*Lp(a) comparison type:* Categorical One effect size reported for the comparison of controls (reference) vs. cases. This was reported as statistically significant difference for the unconditional likelihood estimate (0.0031) suggesting a positive association (same direction) The results show that serum Lp(a) concentration is an independent risk factor for subsequent MI or death from CHD
TNT Study [76] (*n* = 1506) *Study design:* RCT *Follow-up (median):* 4.9 yrs *Population description:*Mixed gender adults ≥ 40 yrs who have experienced major cardiovascular events and are receiving statin treatment *Risk of bias assessment overall* ^a^ *:* High *Funding:* Pharma	*Model:* Cox proportional hazards regression *Variables*: Age, gender and other variables; LDL-C not included *CVD Outcomes:* CHD death; non-fatal, non-procedure-related myocardial infarction; resuscitated cardiac arrest; and fatal or nonfatal stroke. *Lp(a) assay:* Commercial assay; Immunoturbidimetric assay; Isoform dependence - NR/unclear; NR if fresh or frozen samples	*Lp(a) comparison type:* Categorical Three effect sizes reported. Two out of three effect sizes were statistically significant and all were positive association (same direction). One effect size was reported in whole population for Lp(a) with major CV events: HR 1.17, 95 % CI: 1.04 to 1.33. Other significant effect size was reported in subgroup atorvastatin 10 mg QD for Lp(a) with major CV events: HR 1.34, 95 % 1.12 to 1.6. No significant effect in Atorvastatin 80 mg subgroup 1.01 (95 % CI 0.85 to 1.20) Results suggest that higher plasma levels of Lp(a) are independently associated with an increased risk of recurrent events.
Wehinger 1999 [77] (*n* = 2223) *Study design:* Prospective cohort study *Follow-up:* 1 yr *Population description:* Mixed gender adults ≥ 18 yrs successfully treated with intracoronary stent due to symptomatic CAD *Risk of bias assessment overall* ^a^ *:* Moderate *Funding:* NR	*Model:* Log-rank test *Variables*: Age, gender and other variables; LDL-C not included *CVD Outcomes:* Angiographic restenosis *Lp(a) assay:* Isoform independence – NR/unclear; fresh samples	*Lp(a) comparison type:* Categorical Four effect sizes reported. All were NS for the comparison between Lp(a) quintiles (2 to 3) vs. Lp(a) quintile 1. Results suggest that elevated Lp(a) levels did not influence the one-year clinical and angiographic outcome after stent placement. Thrombotic events and measures of restenosis were not adversely affected by the presence of high Lp(a) levels.

^a^ Overall risk of bias as assessed by QUIPS tool [24]

4S Scandinavian Simvastatin Survival Study; ACS acute coronary syndrome; AIM-HIGH Atherothrombosis Intervention in Metabolic Syndrome with low HDL/High Triglycerides: Impact on Global Health Outcomes; CABG coronary artery bypass grafting; CAD coronary artery disease; CHD coronary heart disease; CI confidence interval; CVD cardiovascular disease; dl decilitre; GENERATION Global Evaluation of New Events and Restenosis After Stent Implantation; HDL high-density lipoprotein; HERS Heart and Estrogen/progestin Replacement Study; HR hazard ratio; LDL-C low-density lipoprotein; l litre; LIPID Long-Term Intervention with Pravastatin in Ischaemic Disease; Lp(a) lipoprotein a; max maximum; MACE major coronary events; mg milligram; MI myocardial infarction; min minimum; NR not reported; NSTACS non-ST-segment elevation acute coronary syndrome; PCI percutaneous coronary intervention; PTCA percutaneous transluminal coronary angioplasty; QUIPS Quality In Prognosis Studies; RCT; ref reference; RESEARCH Rapamycin-Eluting Stent Evaluated At Rotterdam Cardiology Hospital; STEMI ST-segment elevation acute myocardial infarction; TG triglyceride; TIA transient ischemic attack; yrs years

The overall quality of the eight prospective cohort studies [45, 68, 69, 71–74, 77], one nested case-control study [75] and five RCTs [13, 66, 67, 70, 76] was mixed, with the risk of bias assessed as moderate in seven studies [13, 66, 72–75, 77] low in two studies [67, 68] and high in two studies [45, 76]. There was insufficient information to make a proper assessment in the remaining three studies due to poor reporting [69–71].

CVD outcomes assessed in the studies were individual and combined outcomes including the following: CHD [70], non-fatal MI [13, 68–70, 75], cardiovascular death [13, 75, 76], [69, 70, 79] stroke [67], MACE [66, 71–74], hospitalisation for angina [68, 69], MI [70, 72, 76] TIA [67] angiographic restenosis [72, 77] and all deaths [45, 66]. Twelve studies used Cox proportional hazards models [13, 67–74, 76, 77, 80], and two studies used logistic regression [66, 75]. All of the studies considered age and gender in their analyses. The other variables used in the analyses differed in type and number across the studies and on occasions the studies reported results for models adjusted for different groups of variables. Only two studies included LDL-C level in their model (HERS [70] and Park 2015 [70, 72]) and binary restenosis and 3 yr adverse clinical outcomes in an Asian population (Lp(a) > 50 mg/dL (versus Lp(a) ≤ 50 mg/dL [reference]: OR 2.88, 95 % CI: 1.37 to 6.07) [72]. In addition, one further study (Kwon 2015 [73]) included the presence of hyperlipidemia (defined as LDL-C ≥ 130 mg/dL) as a variable in the multivariable model. This study also concluded that elevated Lp(a) is associated with worse outcomes (MACE) in type 2 diabetics patients with symptomatic CAD (Tertile 1 [median 4.7 mg/dL; reference] versus Tertile 2 [median 13.5 mg/dL]: HR 1.54, 95 % CI: 0.68 to 3.50 and Tertile 1 [median 4.7 mg/dL; reference] versus Tertile 3 [median 38.8 mg/dL]: HR 2.89, 95 % CI: 1.37 to 6.08).

Five studies (HERS; [70] Ezhov 2014; [68] LIPID; [13] Kwon 2015; [73] and RESEARCH [74]) reported the use of an isoform independent Lp(a) assay. All of these studies concluded that increased baseline [13, 68, 70, 72] or follow-up [13] Lp(a) concentration was a significant and independent risk factor for CVD events including recurrent CHD [70], MACE [68, 73], total CHD events [13] and prognosis after PCI [74]. HERS [70] reported that increased baseline Lp(a) concentrations (≥ 25.4 mg/dL) of Lp(a) were associated with significant and independent increased in CHD (HRs between 1.01 and 1.31). Ezhov 2014 [68] reported that stable CHD patients with Lp(a) ≥30 mg/dL were at a significantly greater risk of cardiovascular death and MI (HR 2.98, 95 % confidence interval [CI]: 1.76 to 5.03) and cardiovascular death, MI, hospitalization for recurrent or unstable angina and repeat revascularization (HR 3.47, 95 % CI: 2.48 to 4.85), than patients with Lp(a) values <30 mg/dL. The LIPID [13] study as reported that increased baseline Lp(a) concentrations were independently associated with an increased risk of total CHD events (*p* < 0.001), total cardiovascular disease events (*p* = 0.002), and coronary events (*p* = 0.03). The authors also reported that the greatest risk occurred at Lp(a) concentrations >73 mg/dL (upper decile) and that an increase in Lp(a) concentration at 1 year was associated with an increased risk of total CHD events and total cardiovascular disease events (both *p* = 0.002). The RESEARCH [74] study concluded that there was a significant and independent association between Lp(a) concentrations before PCI and a higher risk of MACE at 1-year follow-up (HR 3.1, 95 % CI: 1.1 to 8.6 for the highest versus [≥ 65.2 nmol/L] the lowest tertile [<9.8 nmol/L]). However, the authors reported that this association weakened and lost significance with long-term follow-up.

Overall, half of the studies included Lp(a) level as a continuous variable and the other half of the studies used categorical data. Where Lp(a) was assessed as a categorical value, the thresholds for the categories differed between studies. Across all of the 14 studies, 91 effect sizes were reported for the association between Lp(a) and CVD events, 36 were statistically significant, showing a positive association, between increased Lp(a) levels and the risk of CVD events (HRs ranged from 0.75 to 3.7). Only one study reported a negative association (Wehringer 1999 [77]), which was not statistically significant. This study concluded that elevated Lp(a) mass did not influence the one-year clinical and angiographic outcome after stent placement [77].

The remaining 53 analyses suggested that Lp(a) predicted CV outcomes (HRs ranged from 1.21 to 3.7), though not all of the effect sizes were statistically significant. This included results from five studies (4S study; [66] GENERATION; [69] HERS; [70] LIPID; [13, 74] and RESEARCH [74]). Although some non-significant results for subgroup and sensitivity analyses were reported in these five studies, all concluded that high Lp(a) concentrations (range ≥ 25 to 65.2 mg/dL) were significant predictors of CVD events, including in populations of postmenopausal women with CHD [70], patients with previous CHD [66], stable CHD [13], stable and unstable coronary syndromes after coronary stenting [69] and short term progress after PCI [74].

## Discussion

This review presents contemporary evidence examining the extent of relationship between Lp(a) levels and CVD outcomes. The strengths of the review include the adherence to validated rigorous systematic review methodology. This is also one of the first systematic reviews to examine high risk primary prevention patients and secondary prevention populations separately, while only focusing on evidence from multivariable analyses which took into consideration potential confounders.

Our review suggests that evidence is available to support an independent positive association between Lp(a) and the risk of future CVD events both in the general population and in high risk populations, such as those with diabetes, hypertension, or on dialysis. Evidence also exists to support the positive independent association of Lp(a) mass with CVD events in secondary prevention populations. The number of studies for high risk primary prevention populations and secondary prevention populations was limited.

Our findings confirm previous reviews of primary prevention studies; [16, 18] including a review by the Emerging Risk Factors Collaboration published in 2009. This review of 37 prospective studies (*n* = 1,40,956) reported that Lp(a) modelled as continuous and categorical variables, was an independent risk factor for coronary heart disease death, nonfatal MI, and stroke [16]. The review used individual patient data from the included studies and focused on the primary prevention of coronary heart disease, stroke and non-vascular mortality. In comparison, our review was based on study level data and included a much broader range of CVD outcomes and encompasses primary prevention in high risk population and secondary prevention populations.

Our review found four out of 57 studies which concluded that Lp(a) mass was not a predictor of subsequent events. This included three primary prevention studies [33, 39, 51] in the general population and one in a high risk population [58]. The study in a high risk population reported that in stable haemodialysis patients measuring outcomes at two years, Lp(a) mass was related to the development of overall death and cardiovascular death, but suggested that it was involved in an acute phase reaction and successful treatment of the inflammatory condition may improve long-term survival and so explain the lack of an association with mortality in these patients [58]. The primary prevention studies in the general population concluded that there was no overall statistically significant association between Lp(a) mass and the risk of all-cause mortality, cumulative fatal–nonfatal stroke, and cumulative fatal–nonfatal CAD events [51], ischemic stroke in postmenopausal women [39] and peripheral arterial disease in men [33]. This may have been due to inadequate measurement methods for Lp(a) in comparison with other primary prevention studies, although methods were not always clearly described with respect to their isoform independence [39].

With respect to the relationship between Lp(a) mass and the secondary prevention of CVD outcomes, our review found some evidence to support a positive association in agreement with previous reviews in this population [81, 82] including a recent review by O’Donoghue [20]. Unlike our review, O’Donoghue and colleagues had access to individual patient data from three studies (PEACE – Prevention of Events with Angiotensin Converting Enzyme Inhibition; [83] CARE - Cholesterol and Recurrent Event); [84] and PROVE IT–TIMI 22 - Pravastatin or Atorvastatin Evaluation and Infection Therapy–Thrombolysis In Myocardial Infarction 22 [85] trials) and were able to pool these data. When combined with in some cases unpublished data from eight previous studies (FATS - Familial Atherosclerosis Treatment Study; [86] 4S; [66] HERS; [70] GENERATION; [69] Saely 2006; [87] Skinner 1997; [88] Stubbs 1998; [89] and AIM-HIGH [67]) the authors also found a significant association between Lp(a) and the risk of future myocardial infarction, MACE (OR 1.40, 95 % CI: 1.15 to 1.71). This suggests that although our review did not necessarily have access to all of these trial data, similar conclusions were evident.

Only one study [77] in our review concluded that elevated Lp(a) levels were not predictive of CVD events after stent placement. In this case, Lp(a) mass was found not to influence the one-year clinical and angiographic outcome after stent placement, but the study did not use a standardised assay for Lp(a) level determination and may have been confounded by the use of antithrombotic drugs post stent placement [77]. Previous reviews in secondary prevention have similarly reported a positive association between Lp(a) and CVD event for populations including those who have experienced a stroke [81], and patients who have experienced in-stent restenosis after coronary stenting [82]. However, unlike our review these reviews have not focused specifically on multivariable studies to control for confounding factors.

The relationship between Lp(a) mass and stroke is of particular interest clinically and our review suggests that there is evidence to suggest a significant positive relationship between Lp(a) and non-haemorrhagic stroke in high risk [54, 65] and secondary prevention [67] populations, in agreement with the findings of other recent reviews [16]. However, the risk relationship in the general population was not as clearly defined, with the suggestion that an effect is only present in certain subgroups of the population. For instance, Lp(a) appeared to independently predict fatal and non-fatal stroke/TIA in middle-aged men [26], but not for ischemic stroke in postmenopausal women [39]. The effects of Lp(a) levels on stroke including ischemic stroke were not as well investigated however, and there is a need for more well designed studies to look at the specific effects of Lp(a) with regard to the different stroke subtypes including ischemic stroke.

The identification of Lp(a) as a potential risk factor for CVD/CHD risk prompts the question as to whether this would be an appropriate biomarker for risk stratification and screening [90]. To date, RCTs have not been performed in patients with elevated Lp(a) levels that were randomized to a therapy, primarily due to lack of therapeutic agents developed specifically to lower Lp(a). In addition, there is also a lack of clinical trial evidence to show that Lp(a) reduction (independent of effects on LDL-C) lowers CVD event risk. Only half of the studies in our review included LDL-C as a variable in the multivariable model. Despite this, some studies have suggested a genetic basis for a link between Lp(a) and CVD. Evidence from multiple genome wide association [91, 92] and Mendelian randomization studies [93], suggested that LPA gene variants (encoding Lp(a) lipoprotein) were strongly associated with both an increased level of Lp(a) lipoprotein and an increased risk of coronary disease. However, a more recent Mendelian randomization study has suggested that Lp(a) promotes CVD through atherosclerotic stenosis whereas possible prothrombotic effects appear less influential [94]. In addition, evidence suggests that Lp(a)-lowering therapy such as a Lp(a) apheresis with immunabsorption against human apo(a) results in a significant improvement in the stenosis of the coronary arteries without evidence of other major changes in the lipid profile [95].

In addition, Lp(a) may enhance risk discrimination and reclassification; a recent study on the predictive role of Lp(a) in long-term (15 years) CVD outcomes in general community showed that the net reclassification improvement afforded by Lp(a) was as high as 39.6 % in intermediate-risk group and indicated that Lp(a) can modify clinical risk assessment [96, 97]. The 2015 National Lipid Association recommendations for patient-centered management of dyslipidemia suggest that the presence of Lp(a) levels of 50 mg/dL or more may warrant moving a patient into a higher risk category [98]. Similarly, the European Atherosclerosis Society consensus panel recommends screening for elevated Lp(a) in those at intermediate or high CVD/CHD risk and a desirable level <50 mg/dL as a function of global cardiovascular risk [18]. This would also suggest that current and future treatments which reduce Lp(a) levels, such as apheresis [95, 99], antisense therapy which targets Apo(a) [100] and PCSK9 inhibitors [10, 101, 102] could provide additional benefit in the treatment of patients at risk of CVD beyond LDL-C lowering [18], and evidence of such additional benefit beyond LDL-C lowering should be investigated further in ongoing and future trials [103].

The analysis within our review was limited by the inability to carry out statistical pooling/meta-analysis. This was not possible due to the considerable variation in outcomes, modelling and Lp(a) assays used. Little can also be concluded about the concurrent effects of LDL-C and Lp(a) as very few of the included studies considered both LDL-C and Lp(a) as model variables. Poor reporting of study methodology in some of the studies also hampered the conduct of the review, particularly during the study selection process and the risk of bias assessment. In addition, in some cases relevant data for studies were not available from individual study publications and were only available in pooled analyses from groups of trialists, which were not eligible for inclusion in our review.

Issues with Lp(a) measurement were also problematic and hampered interpretation as has been noted by previous review authors [11, 104]. The methods used to measure Lp(a) mass were also poorly reported. At present research suggests that there are no commercially available assays that are completely and truly insensitive to the variability in Lp(a) particle mass, and so the development of assays which are mass-insensitive are key to the future interpretation of Lp(a) risk prediction studies [104]. However, for the purposes of this review we have used the authors’ description to classify whether studies were isoform dependent or independent. In the majority of cases the classification was either not reported or unclear. However, the authors of one study (Cho 2010 [46]) clearly reported that the test used was isoform dependent and 17 studies that the test used was isoform independent (CCHS; [97] Chin-Shan Community Cardiovascular Cohort Study; [42] CHOICE; [53] CHS; [105] D’Angelo 2006; [106] EPIC; [107] Ezhov 2014; [68] FHS; [108] GRIPS; [28] HERS; [70] HPFS; [32] LIPID; [13] Lipid Research Clinics Coronary Primary Prevention trial; [35] NHS; [109] RESEARCH; [74] Saely 2006; [87] and WHS [40]). No obvious differences between these two sets of studies were evident in terms of the significance and direction of effects for the relationship. All of the studies of high risk prevention and secondary prevention population that reported the use of an isoform independent test concluded that Lp(a) was an independent risk factor for CVD events. Future studies should ensure that their methodology, including Lp(a) assay methods is clearly reported, given the potential issues relating to the reliability of Lp(a) measurement and comparability between assays.

## Conclusions

There is evidence to suggest that increased Lp(a) levels are associated with modest increases in the risk of future CVD in both of the lower and higher risk populations reviewed. Therapies that provide Lp(a) lowering in addition to LDL-C lowering such as PCSK9 inhibitors and antisense therapy which targets Apo(a), should be investigated for additional benefit in these populations beyond the expected benefits of the LDL-C lowering.

## Additional files

Additional file 1: Search strategies. (DOCX 25 kb) Additional file 2: Table S1. Summary of study characteristics (60 studies). (DOCX 59 kb) Additional file 3: Table S2. Summary of QUIPS quality assessment domains (60 studies). (DOCX 54 kb)

## Abbreviations

4S: Scandinavian Simvastatin Survival Study
ACS: acute coronary syndrome
AIM-HIGH: Atherothrombosis Intervention in Metabolic Syndrome with low HDL/High Triglycerides: Impact on Global Health Outcomes
Apo(a): apoprotein(a)
ASCVD: atherosclerotic cardiovascular disease
CABG: coronary artery bypass grafting
CAD: coronary artery disease
CD: coronary death
CV: cardiovascular
CHD: coronary heart disease
CHOICE: Choices for Healthy Outcomes in Caring for ESRD
CI: confidence interval
CVD: cardiovascular disease
dl: decilitre
ECG: electrocardiogram
ESRD: end stage renal disease
GENERATION: Global Evaluation of New Events and Restenosis After Stent Implantation
HDL: high-density lipoprotein
HERS: Heart and Estrogen/progestin Replacement Study
HF: heart failure
HR: hazard ratio
ICD: International Classification of Diseases
IHD: ischemic heart disease
JDS: Japan Diabetes Complications Study
LDL-C: low density lipoprotein cholesterol
l: litre
LIPID: Long-Term Intervention with Pravastatin in Ischaemic Disease
Lp(a): lipoprotein(a)
max: maximum
LVH: left ventricular hypertrophy
MACE: major adverse cardiac events
mg: milligram
MI: myocardial infarction
min: minimum
mth: months
NR: not reported
NS: not significant
NSTACS: non-ST-segment elevation acute coronary syndrome
OR: odds ratio
PAD: peripheral arterial disease
PCI: percutaneous coronary intervention
PTCA: percutaneous transluminal coronary angioplasty
QUIPS: Quality In Prognosis Studies
RCT: randomised controlled trial
RESEARCH: Rapamycin-Eluting Stent Evaluated At Rotterdam Cardiology Hospital
STEMI: ST-segment elevation acute myocardial infarction
TIA: transient ischemic attack
TG: triglyceride
TLR: target lesion revascularization
TVR: target vessel revascularization
UK: United Kingdom
USA: United States of America
WHO: World Health Organisation
Yrs: years
