# Supplementary material for: The relationship between Lp(a) and CVD outcomes: a systematic review
Source: Lipids Health Dis. 2016 May 17;15:95. doi: 10.1186/s12944-016-0258-8 (PMC4869344; doi:10.1186/s12944-016-0258-8)
Supplement: Additional file 1: — Search strategies. (DOCX 25 kb) [file 12944_2016_258_MOESM1_ESM.docx]

**Additional file 1: Search strategies**

**Embase (Ovid): 1974 to 2016 April 08**

**Searched: 11.4.16**

**Records found: 1057**

1 lipoprotein A/ (7344)

2 ("lipoprotein a" or lipoproteina or "lipoprotein(a)" or "lp(a)" or "l-pa").ti,ab,ot. (9960)

3 or/1-2 (11507)

4 Random$.tw. or clinical trial$.mp. or exp health care quality/ (3789176)

5 ((longitudinal$ or prospective$) adj2 (study or studies or trial or trials)).ti,ab. (418472)

6 (cohort$ adj2 (study or studies or trial or trials)).ti,ab. (178593)

7 cohort analysis/ (237493)

8 longitudinal study/ (86448)

9 or/4-8 (4232120)

10 animal/ (1751022)

11 animal experiment/ (1921778)

12 (rat or rats or mouse or mice or murine or rodent or rodents or hamster or hamsters or pig or pigs or porcine or rabbit or rabbits or animal or animals or dogs or dog or cats or cow or bovine or sheep or ovine or monkey or monkeys).ti,ab,ot,hw. (6203310)

13 or/10-12 (6203310)

14 exp human/ (17044992)

15 human experiment/ (351051)

16 or/14-15 (17046439)

17 13 not (13 and 16) (4878926)

18 9 not 17 (4042005)

19 3 and 18 (3121)

20 prognosis/ (484906)

21 (predict$ or prognos$ or determinant$ or correlat$ or correlant$ or relation$).ti,ab. (4666723)

22 or/20-21 (4806153)

23 19 and 22 (1064)

24 "2016".yr. (196924)

25 23 not 24 (1057)

**Trial filter:**

Wong SS, Wilczynski NL, Haynes RB. Developing optimal search strategies for detecting clinically sound treatment studies in EMBASE. J Med Libr Assoc 2006;94(1):41-7.

**Based on observational studies filter:**

Scottish Intercollegiate Guidelines Network (SIGN). Search filters: observational studies [Embase (OvidSP)]. Edinburgh: SIGN, Last modified 26/04/13 Available from: <http://www.sign.ac.uk/methodology/filters.html#obs>

**MEDLINE (Ovid): 1946 to March Week 5 2016**

**Searched: 11.4.16**

**Records found: 1716**

1 "Lipoprotein(a)"/ (4470)

2 ("lipoprotein a" or lipoproteina or "lipoprotein(a)" or "lp(a)" or "l-pa").ti,ab,ot. (7546)

3 or/1-2 (8036)

4 randomized controlled trial.pt. or "randomized controlled trials as topic"/ (508916)

5 controlled clinical trial.pt. (90457)

6 random$.ti,ot. (125189)

7 placebo.ab. (157136)

8 drug therapy.fs. (1841827)

9 random$.ab. (710018)

10 trial.ab. (319135)

11 groups.ab. (1379535)

12 ((longitudinal$ or prospective$) adj2 (study or studies or trial or trials)).ti,ab. (278069)

13 exp Cohort Studies/ (1517858)

14 (cohort$ adj2 (study or studies or trial or trials)).ti,ab. (110466)

15 or/4-14 (4711513)

16 exp animals/ not (exp animals/ and humans/) (4221321)

17 15 not 16 (4157137)

18 3 and 17 (3398)

19 exp Prognosis/ (1235990)

20 (predict$ or prognos$ or determinant$ or correlat$ or correlant$ or relation$).ti,ab. (3327306)

21 19 or 20 (4145189)

22 18 and 21 (1716)

23 "2016".yr. (3819)

**24 22 not 23 (1716)**

**Based on trials filter:**

Lefebvre C, Manheimer E, Glanville J. Chapter 6: searching for studies. Box 6.4.c: Cochrane Highly sensitive search strategy for identifying randomized controlled trials in Medline: Sensitivity-maximizing version (2008 version); OVID format. In: Higgins JPT, Green S (editors). Cochrane Handbook for Systematic Reviews of Interventions Version 5.1.0 [updated March 2011]. The Cochrane Collaboration, 2011. Available from [www.cochrane-handbook.org](http://www.cochrane-handbook.org)

**Based on observational studies filter:**

Scottish Intercollegiate Guidelines Network (SIGN). Search filters: observational studies [MEDLINE (OvidSP)]. Edinburgh: SIGN, Last modified 26/04/13 Available from: <http://www.sign.ac.uk/methodology/filters.html#obs>

**MEDLINE In-Process & Other Non-Indexed Citations, MEDLINE Daily Update (Ovid): April 08, 2016**

**Searched: 11.4.16**

**Records found: 43**

1 "Lipoprotein(a)"/ (1)

2 ("lipoprotein a" or lipoproteina or "lipoprotein(a)" or "lp(a)" or "l-pa").ti,ab,ot. (345)

3 or/1-2 (345)

4 randomized controlled trial.pt. or "randomized controlled trials as topic"/ (1296)

5 controlled clinical trial.pt. (58)

6 random$.ti,ot. (19806)

7 placebo.ab. (11595)

8 drug therapy.fs. (2176)

9 random$.ab. (90601)

10 trial.ab. (35736)

11 groups.ab. (160972)

12 ((longitudinal$ or prospective$) adj2 (study or studies or trial or trials)).ti,ab. (27760)

13 exp Cohort Studies/ (2475)

14 (cohort$ adj2 (study or studies or trial or trials)).ti,ab. (16231)

15 or/4-14 (271789)

16 exp animals/ not (exp animals/ and humans/) (2657)

17 15 not 16 (271219)

18 3 and 17 (122)

19 exp Prognosis/ (2281)

20 (predict$ or prognos$ or determinant$ or correlat$ or correlant$ or relation$).ti,ab. (390886)

21 19 or 20 (392330)

22 18 and 21 (54)

23 "2016".yr. (285104)

**24 22 not 23 (43)**

**Based on trials filter:**

Lefebvre C, Manheimer E, Glanville J. Chapter 6: searching for studies. Box 6.4.c: Cochrane Highly sensitive search strategy for identifying randomized controlled trials in Medline: Sensitivity-maximizing version (2008 version); OVID format. In: Higgins JPT, Green S (editors). Cochrane Handbook for Systematic Reviews of Interventions Version 5.1.0 [updated March 2011]. The Cochrane Collaboration, 2011. Available from www.cochrane-handbook.org

**Based on observational studies filter:**

Scottish Intercollegiate Guidelines Network (SIGN). Search filters: observational studies [MEDLINE (OvidSP)]. Edinburgh: SIGN, Last modified 26/04/13 Available from: <http://www.sign.ac.uk/methodology/filters.html#obs>

**Cochrane Database of Systematic Reviews (Wiley): Issue 4 of 12, April 2016**

**Cochrane Central Register of Controlled Trials (Wiley): Issue 3 of 12, March 2016**

**Database of Abstracts of Reviews of Effects (Wiley): Issue 2 of 4, April 2015**

**Date searched: 11.04.16**

**CENTRAL search retrieved 404 records**

**CDSR search retrieved 27 records**

**DARE search retrieved 5 records**

#1 MeSH descriptor: [Lipoprotein(a)] explode all trees 366

#2 "lipoprotein a" or lipoproteina or "lipoprotein(a)" or "lp(a)" or "l-pa" 1178

#3 #1 or #2 1179

#4 MeSH descriptor: [Prognosis] explode all trees 127375

#5 predict* or prognos* or determinant* or correlat* or correlant* or relation* 182799

#6 #4 or #5 271110

**#7 #3 and #6 in Cochrane Reviews (Reviews and Protocols), Other Reviews and Trials 436**

**NIH Clinicaltrials.gov (Internet): up to 11.4.16**

[**http://clinicaltrials.gov/ct2/search/advanced**](http://clinicaltrials.gov/ct2/search/advanced)

**Searched 11.4.16**

| **Search terms** | **Records** |
| --- | --- |
| "lipoprotein a" OR lipoproteina OR "lipoprotein(a)" OR "lp(a)" OR "lp-a" | 157 |
| **Total** | **157** |

**ISRCTN registry (Internet): up to 11.4.16**

[**http://www.isrctn.com/**](http://www.isrctn.com/editAdvancedSearch)

**Searched 11.4.16**

| **Text Search** | **Records** |
| --- | --- |
| "lipoprotein a" OR lipoproteina OR "lipoprotein(a)" OR "lp(a)" OR "lp-a" | 24 |
| **Total** | **24** |

**European Atherosclerosis Society Congress**

**Searched: 11.4.16**

**Total records found: 208**

79th EAS Congress 2011

via Science Direct at: <http://www.sciencedirect.com/science/journal/15675688/12/1>

80th EAS Congress 2012

<http://kenesforms.kenes.com/eas2012/abstracts/>

*81st EAS Congress 2013*

*- abstracts not available online*

82nd EAS Congress 2014

via Science Direct at: <http://www.sciencedirect.com/science/journal/00219150/235/2>

83rd EAS Congress 2015, Glasgow

<http://www.sciencedirect.com/science/journal/00219150/241/1>

|  | **2011** | **2012** | **2013** | **2014** | **2015** | **Total** |
| --- | --- | --- | --- | --- | --- | --- |
| Lipoprotein a (in title) |  | 1 |  |  |  | 1 |
| Lipoproteina (in title) |  | 0 |  |  |  | 0 |
| Lipoprotein(a) (in title) |  | 4 |  |  |  | 4 |
| lp(a) (in title) |  | 4 |  |  |  | 4 |
| l-pa (in title) |  | 0 |  |  |  | 0 |
| "lipoprotein a" or lipoproteina or "lipoprotein(a)" or "lp(a)" or "l-pa" (all fields) | 151 |  |  | 25 | 23 | 199 |
| **Total** | **151** | **9** | **N/A** | **25** | **23** | **208** |

**European Society of Cardiology Congress**

**Searched: 11.4.16**

**Records found: 45**

ESC Congresses 2011-2014

<http://spo.escardio.org/abstract-book/search.aspx>

| **Search term in 'Presentation Title' field only** | **2011** | **2012** | **2013** | **2014** | **TOTAL** |
| --- | --- | --- | --- | --- | --- |
| Lipoprotein a | 0 | 0 | 0 | 0 | 0 |
| Lipoproteina | 0 | 0 | 0 | 0 | 0 |
| Lipoprotein(a) | 2 | 2 | 3 | 4 | 11 |
| lp(a) | 0 | 2 | 1 | 1 | 5 |
| lp-a | 0 | 0 | 0 | 0 | 0 |
| **Total** | **3** | **4** | **4** | **5** | **16** |

ESC Congress 2015

<http://congress365.escardio.org/>

| Lipoprotein | 24 |
| --- | --- |
| Lipoproteina | 0 |
| Lipoprotein(a) | 5 |
| lp(a) | 0 |
| lp-a | 0 |

**American College of Cardiology Annual Scientific Session**

**Searched: 11.4.16**

**Total records found: 25**

2011 Annual Scientific Session

<http://www.abstractsonline.com/EPosterViewer/Home.aspx?Mkey=79E691F3-9BF2-4C60-B958-A23D68B60921>

2012 Annual Scientific Session

<http://www.abstractsonline.com/EPosterViewer/Home.aspx?Mkey=86D3D797-1DEA-4B4D-A5C5-A402B3E95F8C>

2013 Annual Scientific Session

via Science Direct at: <http://www.sciencedirect.com/science/journal/07351097/61/10/supp/S>

2014 Annual Scientific Session

via Science Direct at: <http://www.sciencedirect.com/science/journal/07351097/63/12/supp/S>

2015 Annual Scientific Session

via Science Direct at: <http://www.sciencedirect.com/science/journal/07351097/65/10/supp/S>

| **Search term in Title field only** | **2011** | **2012** | **2013** | **2014** | **2015** | **TOTAL** |
| --- | --- | --- | --- | --- | --- | --- |
| Lipoprotein a | 0 | 0 |  |  |  | 0 |
| Lipoproteina | 0 | 0 |  |  |  | 0 |
| Lipoprotein(a) | 1 | 0 |  |  |  | 1 |
| Lp(a) | 1 | 0 |  |  |  | 1 |
| Lp-a | 0 | 0 |  |  |  | 0 |
| "lipoprotein a" or lipoproteina or "lipoprotein(a)" or "lp(a)" or "l-pa" (all fields) |  |  | 5 | 7 | 11 | 23 |
| **Total** | **2** | **0** | **5** | **7** | **11** | **25** |

**American Heart Association Scientific Sessions**

**Searched: 11.4.16**

**Total records found: 37**

AHA Scientific Sessions 2011-2015

<http://circ.ahajournals.org/search>

| **Search in title** | **Abstract Results 2011** | **Abstract Results 2012** | **Abstract Results 2013** | **Abstract Results 2014** | **Abstract Results 2015** | **TOTAL** |
| --- | --- | --- | --- | --- | --- | --- |
| "lipoprotein a" or lipoproteina or "lipoprotein(a)" or "lp(a)" or "l-pa" | 5 | 8 | 9 | 6 | **9** | **37** |
