# Supplementary material for: The relationship between Lp(a) and CVD outcomes: a systematic review
Source: Lipids Health Dis. 2016 May 17;15:95. doi: 10.1186/s12944-016-0258-8 (PMC4869344; doi:10.1186/s12944-016-0258-8)
Supplement: Additional file 2: Table S1. — Summary of study characteristics (60 studies). (DOCX 59 kb) [file 12944_2016_258_MOESM2_ESM.docx]

**Additional file 2**

**Table S1: Summary of study characteristics (60 studies)**

| **Study Details** | **Population Details** |
| --- | --- |
| **Study Name:** 4S Study  **Study Location:** Europe (Denmark, Finland, Iceland, Norway, Sweden)  **Study Design:** RCT  **Follow-up:** Median: 5.4 years (range 4.9-6.3)  **Lp(a) Assay:** Commercial assay; Radioimmunoassay**;** Isoform dependence - NR/unclear; frozen samples  **CVD outcomes:** Death of any cause and major coronary events (fatal or nonfatal definite or probable acute MI, sudden cardiac death or resuscitated cardiac arrest, or silent MI) | **Gender:** Mixed male and female  **Age:** Adults ≥ 18 yrs  **Ethnicity:** NR/unclear  **Diabetic status:** Mixed population of participants with and without Type 2 diabetes  **Hypertension status:** Mixture of hypertensive and non-hypertensive patients  **Smoking status:** Mixed smoking status  **Baseline CVD Risk:** All participants have previous known CVD events (Angina only (20.9%); MI only (63.2%); Both angina and MI (16.8%)) |
| **Study Name:** Agewall 2002  **Study Location:** Europe (Sweden)  **Study Design:** Prospective cohort study  **Follow-up:** 3.0 ± 0.6 years  **Lp(a) Assay:** Commercial assay; Radioimmunoassay**;** Isoform dependence - NR/unclear; not clear if frozen or fresh samples  **CVD outcomes:** Non-fatal MI or a coronary death | **Gender:** Males only  **Age:** Elderly 50yrs+  **Ethnicity:** NR/unclear  **Diabetic status:** Mixed population of participants with and without Type 2 diabetes  **Hypertension status:** NR/unclear  **Smoking status:** Mixed smoking status  **Baseline CVD Risk:** Participants had mixed CVD event history (Coronary artery disease and/or ECG major changes (23%)) |
| **Study Name:** AIM–HIGH  **Study Location:** North America (USA; Canada)  **Study Design:** RCT  **Follow-up:** Mean follow-up of 3yrs at time of trial termination  **Lp(a) Assay:** In-house assay; Immunochemical assay**;** Isoform dependence - NR/unclear; not clear if frozen or fresh samples  **CVD outcomes:** Ischemic stroke (acute vascular event with focal neurological signs lasting >24 hrs, without evidence of primary intracranial haemorrhage); and Ischemic stroke or TIA (focal symptoms of a presumed ischemic basis lasting <24 hrs). | **Gender:** Mixed male and female  **Age:** Middle aged 40yrs+ (≥ 45yrs)  **Ethnicity:** NR/unclear  **Diabetic status:** Mixed population of participants with and without Type 2 diabetes  **Hypertension status:** Mixture of hypertensive and non-hypertensive patients  **Smoking status:** NR/unclear  **Baseline CVD Risk:** All participants have previous known CVD events (MI (56.3%); Stroke/TIA (21%); AF (7.4%); PVD (13.6%)) |
| **Study Name:** ARIC  **Study Location:** North America (USA)  **Study Design:** Prospective cohort study  **Follow-up:** 13.5 yrs  **Lp(a) Assay:** In-house assay; Immunochemical assay**;** Isoform dependence – NR/unclear  **CVD outcomes:** Ischemic stroke; all hospitalisations and deaths; CHD events with and without stroke. | **Gender:** Mixed male and female  **Age:** Middle aged 40yrs+ (45-64 yrs)  **Ethnicity:** Mixed  **Diabetic status:** Mixed population of participants with and without Type 2 diabetes  **Hypertension status:** Mixture of hypertensive and non-hypertensive patients  **Smoking status:** Mixed smoking status  **Baseline CVD Risk:** Participants had mixed CVD event history (NR) |
| **Study Name:** Bruneck  **Study Location:**  (Italy)  **Study Design:** Prospective cohort study  **Follow-up:** 10 yrs  **Lp(a) Assay:** NR/unclear; Isoform dependence - NR/unclear  **CVD outcomes:** Incident MI, stroke, and TIA; CVD (fatal and non-fatal MI, fatal and non-fatal ischemic stroke, TIA, and symptomatic PAD); CVD death (from ischemic stroke, MI, sudden cardiac death, or AAA rupture), acute CAD (consisting of nonfatal MI, new-onset unstable angina or new-onset severe angina, and acute coronary interventions), and ischemic stroke. | **Gender:** Mixed male and female  **Age:** Middle aged 40yrs+ (40-79 yrs)  **Ethnicity:** Mixed  **Diabetic status:** Mixed population of participants with and without Type 2 diabetes  **Hypertension status:** Mixture of hypertensive and non-hypertensive patients  **Smoking status:** Mixed smoking status  **Baseline CVD Risk:** Participants had mixed CVD event history (Pre-existent CVD (previous fatal and nonfatal myocardial infarction, fatal and nonfatal ischemic stroke, transient ischemic attacks, revascularization procedures, and symptomatic peripheral artery disease) (10.1%)) |
| **Study Name:** BUPA Study  **Study Location:** Europe (UK)  **Study Design:** Prospective cohort study  **Follow-up:** 13 yrs  **Lp(a) Assay:** Details NR/unclear; Isoform dependence - NR/unclear  **CVD outcomes:** Death from ruptured AAA; death from IHD. | **Gender:** Males only  **Age:** Middle aged 40yrs+ (35-64 yrs)  **Ethnicity:** NR/unclear  **Diabetic status:** NR/unclear  **Hypertension status:** NR/unclear  **Smoking status:** NR/unclear  **Baseline CVD Risk:** NR/unclear (NR) |
| **Study Name:** Caaerphilly Study  **Study Location:** Europe (UK)  **Study Design:** Prospective cohort study  **Follow-up:** 9 yrs  **Lp(a) Assay:** Commercial assay; Radioimmunoassay**;** Isoform dependence - NR/unclear  **CVD outcomes:** Major ischemic heart disease (IHD) which included fatal IHD (ICD 410-414), non-fatal MI (WHO criteria), and ECG evidence of MI at follow-up | **Gender:** Males only  **Age:** Middle aged 40yrs+ (45 - 59 yrs)  **Ethnicity:** White  **Diabetic status:** NR/unclear  **Hypertension status:** NR/unclear  **Smoking status:** Mixed smoking status  **Baseline CVD Risk:** NR/unclear (NR) |
| **Study Name:** CCHS  **Study Location:** Europe (Denmark)  **Study Design:** Prospective cohort study  **Follow-up:** 17 yrs  **Lp(a) Assay:** In-house assay; Immunoturbidimetric assay**;** Isoform dependence - isoform independent  **CVD outcomes:** MI and CHD. International Classification of Diseases, 8th edition (ICD-8), codes 410 to 414 and 410 respectively, and 10th edition (ICD-10), codes I20 to I25 and I21 to I22, respectively) | **Gender:** Mixed male and female  **Age:** Adults ≥ 18 yrs (20-80+ yrs)  **Ethnicity:** White  **Diabetic status:** Mixed population of participants with and without Type 2 diabetes  **Hypertension status:** NR/unclear  **Smoking status:** Mixed smoking status  **Baseline CVD Risk:** All participants have no previous known CVD events (All patients had to have no prior history of CHD) |
| **Study Name:** CGPS/CCHS  **Study Location:** Europe (Denmark)  **Study Design:** Prospective cohort study  **Follow-up:** Followed up to May 2011 which varied up to 10yrs follow-up  **Lp(a) Assay:** Commercial assay; Immunoturbidimetric assay**;** Isoform dependence - isoform independent and dependent  **CVD outcomes:** Ischemic heart disease (International Classification of Diseases, 8th edition (ICD-8), codes 410 to 414, and 10th edition (ICD-10), codes I20 to I25) and MI (International Classification of Diseases, 8th edition (ICD-8), code 410, and 10th edition (ICD-10), codes I21 to I22) | **Gender:** Mixed male and female  **Age:** Adults ≥ 18 yrs (20-100 yrs)  **Ethnicity:** White  **Diabetic status:** Mixed population of participants with and without Type 2 diabetes  **Hypertension status:** Mixture of hypertensive and non-hypertensive patients  **Smoking status:** Mixed smoking status  **Baseline CVD Risk:** NR/unclear (NR) |
| **Study Name:** Chin-Shan Community Cardiovascular Cohort Study  **Study Location:** Asia (Taiwan)  **Study Design:** Prospective cohort study  **Follow-up:** Median 13.6 yrs  **Lp(a) Assay:** Commercial assay; Immunochemical assay**;** Isoform dependence - isoform independent  **CVD outcomes:** | **Gender:** Mixed male and female  **Age:** Adults ≥ 18 yrs (35 - 97 yrs)  **Ethnicity:** Other (Taiwanese)  **Diabetic status:** Mixed population of participants with and without Type 2 diabetes  **Hypertension status:** Mixture of hypertensive and non-hypertensive patients  **Smoking status:** Mixed smoking status  **Baseline CVD Risk:** Participants had mixed CVD event history (NR) |
| **Study Name:** Cho 2010  **Study Location:** Asia (Korea)  **Study Design:** Prospective cohort study  **Follow-up:** 1 yr  **Lp(a) Assay:** Commercial assay; Immunonephelometric assay**;** Isoform dependence - isoform dependent  **CVD outcomes:** MACE (cardiac death, non-cardiac death, non-fatal MI, repeat PCI, and CABG) | **Gender:** Mixed male and female  **Age:** Adults ≥ 18 yrs  **Ethnicity:** Other (Korean)  **Diabetic status:** Mixed population of participants with and without Type 2 diabetes  **Hypertension status:** Mixture of hypertensive and non-hypertensive patients  **Smoking status:** Mixed smoking status  **Baseline CVD Risk:** All participants have previous known CVD events (Acute myocardial infarction (100%)) |
| **Study Name:** CHOICE  **Study Location:** North America (USA)  **Study Design:** Prospective cohort study  **Follow-up:** Median 27.4 mths  **Lp(a) Assay:** In-house assay; Immunochemical assay**;** Isoform dependence - isoform independent  **CVD outcomes:** ASCVD (MI, cardiac revascularisation procedure, stroke, carotid endarterectomy, extremity gangrene or peripheral revascularisation procedure, limb amputation, or AAA repair) | **Gender:** Mixed male and female  **Age:** Adults ≥17yrs  **Ethnicity:** Mixed  **Diabetic status:** Mixed population of participants with and without Type 2 diabetes  **Hypertension status:** NR/unclear  **Smoking status:** NR/unclear  **Baseline CVD Risk:** NR/unclear (NR) |
| **Study Name:** CHS  **Study Location:** North America (USA)  **Study Design:** Prospective cohort study  **Follow-up:** Median 7.4 yrs  **Lp(a) Assay:** In-house assay; Immunochemical assay**;** Isoform dependence - isoform independent  **CVD outcomes:** Stroke (cerebrovascular accident or TIA); death due to vascular disease (cerebrovascular events, atherosclerosis including PAD, CHD, and other CV causes); all-cause death; and CHD (angina, MI, coronary angioplasty, or bypass surgery). | **Gender:** Mixed male and female  **Age:** Elderly 65yrs +  **Ethnicity:** White  **Diabetic status:** Mixed population of participants with and without Type 2 diabetes  **Hypertension status:** Mixture of hypertensive and non-hypertensive patients  **Smoking status:** Mixed smoking status  **Baseline CVD Risk:** Participants had mixed CVD event history (History of stroke (3%); family history of MI (30.4%)) |
| **Study Name:** Cleveland Clinic Hemodialysis Cohort  **Study Location:** North America (USA)  **Study Design:** Prospective cohort study  **Follow-up:** 4 yrs  **Lp(a) Assay:** In-house assay; Radioimmunoassay**;** Isoform dependence - NR/unclear  **CVD outcomes:** CV death; atherosclerotic events (non-haemorrhagic cerebral infarction, MI, CABG, percutaneous transluminal angioplasty of coronary arteries, surgery for carotid, aortoiliac or lower-extremity atherosclerosis, or fatal CVD event). | **Gender:** Mixed male and female  **Age:** Adults ≥ 18 yrs  **Ethnicity:** Mixed  **Diabetic status:** Mixed population of participants with and without Type 2 diabetes  **Hypertension status:** Mixture of hypertensive and non-hypertensive patients  **Smoking status:** Mixed smoking status  **Baseline CVD Risk:** Participants had mixed CVD event history (Previous event (27%)) |
| **Study Name:** D'Angelo 2006  **Study Location:** Europe (Italy)  **Study Design:** Prospective cohort study  **Follow-up:** Median 6.3 yrs  **Lp(a) Assay:** Commercial assay; Immunochemical assay**;** Isoform dependence - isoform independent  **CVD outcomes:** All cause death; CHD and stroke death defined using the International Classification of Diseases, Eight and Ninth Revision, codes 410-414 (coronary heart disease, CHD), 430-438 (stroke). | **Gender:** Mixed male and female  **Age:** Elderly 60yrs+ (65 - 87 yrs)  **Ethnicity:** NR/unclear  **Diabetic status:** Mixed population of participants with and without Type 2 diabetes  **Hypertension status:** Mixture of hypertensive and non-hypertensive patients  **Smoking status:** Mixed smoking status  **Baseline CVD Risk:** Participants had mixed CVD event history (History of ischemic heart disease or cardiovascular disease (17.9%)) |
| **Study Name:** Diamant Alpin Collaborative Dialysis Cohort  **Study Location:** Europe (Switzerland, France Italy)  **Study Design:** Prospective cohort study  **Follow-up:** 2 yrs  **Lp(a) Assay:** Immunoturbidimetric assay; Isoform dependence - NR/unclear  **CVD outcomes:** Cardiac events (myocardial infarction, de novo angina pectoris or coronary revascularization, ischemic stroke, or peripheral arterial occlusive disease) and cardiac death (due to cardiac arrhythmia, myocardial infarction, or heart failure) | **Gender:** Mixed male and female  **Age:** Adults 18 yrs+ (22-92 yrs)  **Ethnicity:** NR  **Diabetic status:** Mixed population of participants with and without Type 2 diabetes  **Hypertension status:** Mixture of hypertensive and non-hypertensive patients  **Smoking status:** Mixed smoking status  **Baseline CVD Risk:** Participants had mixed CVD event history |
| **Study Name:** Edinburgh Artery Study  **Study Location:** Europe (UK)  **Study Design:** Prospective cohort study  **Follow-up:** 5 yrs  **Lp(a) Assay:** Commercial assay; Immunochemical assay**;** Isoform dependence – NR/unclear  **CVD outcomes:** MI (fatal, non-fatal and silent); stroke (fatal and non-fatal); PAD (new diagnosis of intermittent claudication on WHO questionnaire); CVD mortality; new angina pectoris. | **Gender:** Mixed male and female  **Age:** Elderly 50yrs+ (55-74 yrs)  **Ethnicity:** White  **Diabetic status:** Mixed population of participants with and without Type 2 diabetes  **Hypertension status:** Mixture of hypertensive and non-hypertensive patients  **Smoking status:** Mixed smoking status  **Baseline CVD Risk:** Participants had mixed CVD event history (NR) |
| **Study Name:** EPIC - Norfolk Cohort  **Study Location:** Europe (UK)  **Study Design:** Nested case control study  **Follow-up:** 6 yrs  **Lp(a) Assay:** In-house assay; Immunochemical assay**;** Isoform dependence - NR/unclear  **CVD outcomes:** CAD (International Classification of Diseases 9th Revision codes 410 to 414). | **Gender:** Mixed male and female  **Age:** Middle aged 40yrs+ (45-79 yrs)  **Ethnicity:** NR/unclear  **Diabetic status:** Mixed population of participants with and without Type 2 diabetes  **Hypertension status:** NR/unclear  **Smoking status:** Mixed smoking status  **Baseline CVD Risk:** All participants have no previous known CVD events (No history of heart attack or stroke) |
| **Study Name:** Ezhov 2014  **Study Location:** Asia (Russia)  **Study Design:** Prospective cohort study  **Follow-up:** 15 years: mean of 8.5 ± 3.5 yrs (range 0.9 -15.0 yrs)  **Lp(a) Assay:** In-house assay; Immunochemical assay**;** Isoform dependence - isoform independent  **CVD outcomes:** Non-fatal MI; CV death | **Gender:** Mixed male and female  **Age:** Adults ≥ 18 yrs (29-80yrs)  **Ethnicity:** NR/unclear  **Diabetic status:** Mixed population of participants with and without Type 2 diabetes  **Hypertension status:** Mixture of hypertensive and non-hypertensive patients  **Smoking status:** Mixed smoking status  **Baseline CVD Risk:** Participants had mixed CVD event history (Positive family history of CHD (38%); pre-existing MI (80%); and severe angina pectoris class IIIeIV according to Canadian Cardiovascular Society (83%)) |
| **Study Name:** FHS  **Study Location:** North America (USA)  **Study Design:** Prospective cohort study  **Follow-up:** At or before exam 5 (through 1995).  **Lp(a) Assay:** Commercial assay; Isoform dependence - isoform independent  **CVD outcomes:** CHD (angina pectoris, MI, coronary insufficiency and coronary death); CHD (MI, sudden CHD death, and non-sudden CHD death). | **Gender:** Mixed male and female  **Age:** Adults ≥ 18 yrs  **Ethnicity:** White  **Diabetic status:** NR/unclear  **Hypertension status:** NR/unclear  **Smoking status:** Mixed smoking status  **Baseline CVD Risk:** Participants had mixed CVD event history (NR) |
| **Study Name:** FINRISK '92 Hemostasis Study  **Study Location:** Europe (Finland)  **Study Design:** Prospective cohort study  **Follow-up:** 9.25 yrs  **Lp(a) Assay:** Commercial assay; Radioimmunoassay**;** Isoform dependence - isoform dependent  **CVD outcomes:** Any CVD event (non-fatal strokes, fatal strokes, non-fatal CHD events, CHD deaths and revascularization) | **Gender:** Mixed male and female  **Age:** Middle aged 40yrs+ (45-64 yrs)  **Ethnicity:** NR/unclear  **Diabetic status:** Mixed population of participants with and without Type 2 diabetes  **Hypertension status:** Mixture of hypertensive and non-hypertensive patients  **Smoking status:** Mixed smoking status  **Baseline CVD Risk:** Participants had mixed CVD event history (No known CVD at baseline (94.1%); CVD at baseline (5.9%)) |
| **Study Name:** Fletcher Challenge Study  **Study Location:** Australia and Oceania (New Zealand)  **Study Design:** Nested case control study  **Follow-up:** Median: 5.5 years  **Lp(a) Assay:** Commercial assay; Immunochemical assay**;** Isoform dependence - NR/unclear  **CVD outcomes:** CHD (fatal coronary event or non-fatal hospital diagnosed MI) | **Gender:** Mixed male and female  **Age:** Adults ≥ 18 yrs (about 50% were below 45yrs and 10% aged 65yrs or more)  **Ethnicity:** NR/unclear  **Diabetic status:** NR/unclear  **Hypertension status:** NR/unclear  **Smoking status:** Mixed smoking status  **Baseline CVD Risk:** NR/unclear (NR) |
| **Study Name:** GENERATION  **Study Location:** NR/unclear (NR)  **Study Design:** Prospective cohort study  **Follow-up:** 1.84 yrs  **Lp(a) Assay:** Commercial assay; isoform dependence - NR/unclear  **CVD outcomes:** Composite of cardiac death, non-nfatal MI or rehospitalisation for rest unstable angina | **Gender:** Mixed male and female  **Age:** Adults ≥ 18 yrs  **Ethnicity:** NR/unclear  **Diabetic status:** Mixed population of participants with and without Type 2 diabetes  **Hypertension status:** Mixture of hypertensive and non-hypertensive patients  **Smoking status:** Mixed smoking status  **Baseline CVD Risk:** Participants had mixed CVD event history (History of cerebrovascular disease or peripheral vascular disease (7.9%)) |
| **Study Name:** GRIPS  **Study Location:** Europe (Germany)  **Study Design:** Prospective cohort study  **Follow-up:** 5 yrs  **Lp(a) Assay:** Commercial assay; Immunochemical assay**;** Isoform dependence - isoform independent  **CVD outcomes:** Sudden coronary death; definite fatal MI; definite non-fatal MI | **Gender:** Males only  **Age:** Middle aged 40yrs+ (40-59.9 yrs)  **Ethnicity:** NR/unclear  **Diabetic status:**  **Hypertension status:**  **Smoking status:**  **Baseline CVD Risk:**  All participants have no previous known CVD events |
| **Study Name:** HERS  **Study Location:** North America (USA)  **Study Design:** RCT  **Follow-up:** 4.1 yrs  **Lp(a) Assay:** Commercial assay; Immunochemical assay**;** Isoform dependence - isoform independent  **CVD outcomes:** CHD (non-fatal MI and CHD death); MI; CHD death; CABG/PTCA; unstable angina. | **Gender:** Females only  **Age:** Elderly 50yrs+ (55-79 yrs)  **Ethnicity:** White  **Diabetic status:** Mixed population of participants with and without Type 2 diabetes  **Hypertension status:** Mixture of hypertensive and non-hypertensive patients  **Smoking status:** Mixed smoking status  **Baseline CVD Risk:** All participants have previous known CVD events (Coronary heart disease (100%)) |
| **Study Name:** HHS  **Study Location:** Europe (Finland)  **Study Design:** Nested case control study  **Follow-up:** 8.5 yrs  **Lp(a) Assay:** Commercial assay; Radioimmunoassay**;** Isoform dependence - NR/unclear  **CVD outcomes:** Fatal and non-fatal MI or cardiac death | **Gender:** Males only  **Age:** Middle aged 40yrs+ (40-55 yrs)  **Ethnicity:** NR/unclear  **Diabetic status:** Mixed population of participants with and without Type 2 diabetes  **Hypertension status:** Mixture of hypertensive and non-hypertensive patients  **Smoking status:** Mixed smoking status  **Baseline CVD Risk:** NR/unclear (Devoid of coronary heart disease (100%)) |
| **Study Name:** HPFS  **Study Location:** North America (USA)  **Study Design:** Nested case control study  **Follow-up:** 6 yrs  **Lp(a) Assay:** In-house assay; Immunoturbidimetric assay**;** Isoform dependence - isoform independent  **CVD outcomes:** Non-fatal MI (meets WHO criteria - symptoms plus either diagnostic ECG changes or elevated levels of cardiac enzymes) or fatal CHD (defined as fatal MI confirmed by hospital records or on autopsy or if CHD was listed as the cause of death on the death certificate) | **Gender:** Males only  **Age:** Middle aged 40yrs+ (40-75 yrs)  **Ethnicity:** White  **Diabetic status:** Mixed population of participants with and without Type 2 diabetes  **Hypertension status:** Mixture of hypertensive and non-hypertensive patients  **Smoking status:** Mixed smoking status  **Baseline CVD Risk:** All participants have no previous known CVD events (Inclusion criteria exclude previous CVD events) |
| **Study Name:** HPFS and NHS Study  **Study Location:** North America (USA)  **Study Design:** Nested case control study  **Follow-up:** NR  **Lp(a) Assay:** In-house assay; Immunochemical assay**;** Isoform dependence - NR/unclear  **CVD outcomes:** PAD (arterial disease below the aortic bifurcation, e.g. excluding AAA and renal artery stenosis). | **Gender:** Females only  **Age:** Adults ≥ 18 yrs (30-55yrs)  **Ethnicity:** White  **Diabetic status:** Mixed population of participants with and without Type 2 diabetes  **Hypertension status:** Mixture of hypertensive and non-hypertensive patients  **Smoking status:** Mixed smoking status  **Baseline CVD Risk:** All participants have no previous known CVD events (Inclusion criteria state no previous CVD events) |
| **Study Name:** Ikenaga 2011  **Study Location:** Asia (Japan)  **Study Design:** Prospective cohort study  **Follow-up:** 5 yrs  **Lp(a) Assay:** Commercial assay; Immunoturbidimetric assay**;** Isoform dependence - NR/unclear  **CVD outcomes:** MACE: cardiac death, MI and/or revascularisation for new lesions | **Gender:** Mixed male and female  **Age:** Adults ≥ 18 yrs  **Ethnicity:** Other (Japanese)  **Diabetic status:** Mixed population of participants with and without Type 2 diabetes  **Hypertension status:** Mixture of hypertensive and non-hypertensive patients  **Smoking status:** Mixed smoking status  **Baseline CVD Risk:** All participants have previous known CVD events (Acute MI (100%)) |
| **Study Name:** ILSA  **Study Location:** Europe (Italy)  **Study Design:** Prospective cohort study  **Follow-up:** 6.3 yrs (median)  **Lp(a) Assay:** Commercial assay; Isoform dependence - NR/unclear  **CVD outcomes:** All cause death; fatal and non-fatal stroke events; fatal and non-fatal CAD events; stroke (ischemic, haemorrhagic or TIA) | **Gender:** Mixed male and female  **Age:** Elderly 65yrs +  **Ethnicity:** NR/unclear  **Diabetic status:** Mixed population of participants with and without Type 2 diabetes  **Hypertension status:** Mixture of hypertensive and non-hypertensive patients  **Smoking status:** Mixed smoking status  **Baseline CVD Risk:** Participants had mixed CVD event history (CAD (15.3%); Stroke (5.9%); PAD (2.7%)) |
| **Study Name:** InCHIANTI Study  **Study Location:** Europe (Italy)  **Study Design:** Prospective cohort study  **Follow-up:** 6 yrs  **Lp(a) Assay:** Commercial assay; Immunochemical assay; Isoform dependence - NR/unclear  **CVD outcomes:** PAD | **Gender:** Mixed male and female  **Age:** Elderly 60yrs+  **Ethnicity:** White  **Diabetic status:** Mixed population of participants with and without Type 2 diabetes  **Hypertension status:** Mixture of hypertensive and non-hypertensive patients  **Smoking status:** Mixed smoking status  **Baseline CVD Risk:** Participants had mixed CVD event history |
| **Study Name:** JDCS  **Study Location:** Asia (Japan)  **Study Design:** RCT  **Follow-up:** 7.8 yrs (median)  **Lp(a) Assay:** Details NR/unclear; Isoform dependence - NR/unclear  **CVD outcomes:** Stroke (ischemic, hemorrhagic or TIA); CHD. | **Gender:** Mixed male and female  **Age:** Middle aged 40yrs+ (40-70yrs)  **Ethnicity:** Other (Japanese)  **Diabetic status:** All participants have Type 2 diabetes  **Hypertension status:** Mixture of hypertensive and non-hypertensive patients  **Smoking status:** Mixed smoking status  **Baseline CVD Risk:** All participants have no previous known CVD events (NR) |
| **Study Name:** JUPITER  **Study Location:** Worldwide (USA, Argentina, Belgium, Brazil, Bulgaria, Canada, Chile, Colombia, Costa Rica, Denmark, El Salvador, Estonia, Germany, Israel, Mexico, Netherlands, Norway, Panama, Poland, Puerto Rico, Romania, Russia, South Africa, Switzerland, United Kingdom , Uruguay, Venezuela)  **Study Design:** RCT  **Follow-up:** 2yrs (median)  **Lp(a) Assay:** Commercial assay; Isoform dependence - NR/unclear  **CVD outcomes:** CVD (incident MI, stroke, hospitalization for unstable angina, arterial revascularization, or CV death); all-cause death. | **Gender:** Mixed male and female  **Age:** Elderly 50yrs+  **Ethnicity:** White  **Diabetic status:** No participants have Type 2 diabetes  **Hypertension status:** NR/unclear  **Smoking status:** Mixed smoking status  **Baseline CVD Risk:** All participants have no previous known CVD events (NR) |
| **Study Name:** Kim 2015  **Study Location:** Asia (Korea)  **Study Design:** Prospective cohort study  **Follow-up:** Median 5.5 yrs  **Lp(a) Assay:** NR**;** Isoform independence - NR/unclear  **CVD outcomes:** CVD death and cerebrovascular death | **Gender:** Mixed male and female  **Age:** Adults ≥ 18 yrs  **Ethnicity:** Other (Korean)  **Diabetic status:** NR/unclear  **Hypertension status:** NR/unclear  **Smoking status:** NR/unclear  **Baseline CVD Risk:** NR/unclear |
| **Study Name:** Koda 1999[^56^](#_ENREF_56)  **Study Location:** Asia (Japan)  **Study Design:** Prospective cohort study  **Follow-up:** 2.3 yrs  **Lp(a) Assay:** Immunoturbidimetric assay**;** Isoform dependence - NR/unclear  **CVD outcomes:** All deaths and CV deaths | **Gender:** Mixed male and female  **Age:** Adults ≥ 18 yrs  **Ethnicity:** Other (Japanese)  **Diabetic status:** Mixed population of participants with and without Type 2 diabetes  **Hypertension status:** NR/unclear  **Smoking status:** NR/unclear  **Baseline CVD Risk:** NR/unclear |
| **Study Name:** Konishi 2013  **Study Location:** Asia (Japan)  **Study Design:** Prospective cohort study  **Follow-up:** 4.7 yrs (median)  **Lp(a) Assay:** Details NR/unclear; Isoform dependence - NR/unclear  **CVD outcomes:** All-cause death and acute coronary syndrome. | **Gender:** Males only  **Age:** Adults ≥ 18 yrs  **Ethnicity:** Other (Japanese)  **Diabetic status:** Mixed population of participants with and without Type 2 diabetes  **Hypertension status:** Mixture of hypertensive and non-hypertensive patients  **Smoking status:** Mixed smoking status  **Baseline CVD Risk:** All participants have previous known CVD events (Percutaneous coronary intervention (100%)) |
| **Study Name:** Kwon 2015  **Study Location:** Asia (South Korea)  **Study Design:** Prospective cohort study  **Follow-up:** 4.4 (SD 2.6) yrs  **Lp(a) Assay:** Commercial assay; latex agglutination; Isoform independent  **CVD outcomes:** MACE (cardiac death and non-fatal MI) | **Gender:** Mixed male and female  **Age:** Adults ≥ 18 yrs  **Ethnicity:** Other (Korean)  **Diabetic status:** All participants have Type 2 diabetes and evidence of CAD  **Hypertension status:** Mixture of hypertensive and non-hypertensive patients  **Smoking status:** Mixed smoking status  **Baseline CVD Risk:** All participants have previous known CVD events. All have symptomatic CAD including: IHD (21%); stable angina (43.5%); unstable angina (26%); obstructive CAD (78.5%); NSTEMI (5.8%); STEMI (3.9%); and revascularisation (59.3%) |
| **Study Name:** LIPID  **Study Location:** Australia and Oceania (Australia; New Zealand)  **Study Design:** RCT  **Follow-up:** 6 yrs (median)  **Lp(a) Assay:** Commercial assay; Immunochemical assay**;** Isoform dependence - isoform independent  **CVD outcomes:** CHD (non-fatal MI and CHD death); non-fatal MI; unstable angina; coronary revascularisation; CVD (CVD death, non-fatal MI, non-haemorrhagic stroke, unstable angina, and coronary revascularisation); CHD (non-fatal MI, CHD death, unstable angina, and coronary revascularisation) | **Gender:** Mixed male and female  **Age:** Adults ≥ 18 yrs (31-75 yrs)  **Ethnicity:** NR/unclear  **Diabetic status:** Mixed population of participants with and without Type 2 diabetes  **Hypertension status:** Mixture of hypertensive and non-hypertensive patients  **Smoking status:** Mixed smoking status  **Baseline CVD Risk:** All participants have previous known CVD events (PTCA only (11%); CABG onl (25%); PTCA and CABG (3%); single MI (53%); multiple MI (11%)) |
| **Study Name:** Lipid Research Clinics Coronary Primary Prevention Trial  **Study Location:** North America (NR)  **Study Design:** Nested case control study  **Follow-up:** 7 to 10 yrs  **Lp(a) Assay:** Commercial assay; Immunochemical assay**;** Isoform dependence - isoform independent  **CVD outcomes:** CHD (death and non-fatal MI) | **Gender:** Males only  **Age:** Middle aged 40yrs+ (35-59 yrs)  **Ethnicity:** White  **Diabetic status:** No participants have Type 2 diabetes  **Hypertension status:** NR/unclear  **Smoking status:** Mixed smoking status  **Baseline CVD Risk:** NR/unclear (Any clinical manifestation of coronary heart disease (0%)) |
| **Study Name:** MEGA Study  **Study Location:** Asia (Japan)  **Study Design:** RCT  **Follow-up:** 5.3yrs  **Lp(a) Assay:** Details NR/unclear; Isoform dependence - NR/unclear  **CVD outcomes:** All strokes; ischemic strokes | **Gender:** Mixed male and female  **Age:** Middle aged 40yrs+ (40-70yrs)  **Ethnicity:** NR/unclear  **Diabetic status:** Mixed population of participants with and without Type 2 diabetes  **Hypertension status:** Mixture of hypertensive and non-hypertensive patients  **Smoking status:** Mixed smoking status  **Baseline CVD Risk:** All participants have no previous known CVD events (NR) |
| **Study Name:** Park 2015  **Study Location:** Asia (Korea)  **Study Design:** Prospective PCI registry  **Follow-up:** 3 yrs  **Lp(a) Assay:** Details – NR/unclear**;** Isoform independence - NR/unclear  **CVD outcomes:** CVD death and cerebrovascular death | **Gender:** Mixed male and female  **Age:** Adults ≥ 18 yrs  **Ethnicity:** Other (Korean)  **Diabetic status:** Mixed population of participants with and without Type 2 diabetes  **Hypertension status:** Mixture of hypertensive and non-hypertensive patients  **Smoking status:** Mixed smoking status  **Baseline CVD Risk:** All participants have no previous known CVD events (PCI with DES) |
| **Study Name:** PHS  **Study Location:** North America (USA)  **Study Design:** Nested case control study  **Follow-up:** 9yrs  **Lp(a) Assay:** Commercial assay; Immunonephelometric assay**;** Isoform dependence - NR/unclear  **CVD outcomes:** PAD (intermittent claudication or peripheral artery surgery); future angina (angina and additional evidence of severe atherosclerotic disease) | **Gender:** Males only  **Age:** Middle aged 40yrs+ (40 to 84yrs)  **Ethnicity:** NR/unclear  **Diabetic status:** Mixed population of participants with and without Type 2 diabetes  **Hypertension status:** Mixture of hypertensive and non-hypertensive patients  **Smoking status:** Mixed smoking status  **Baseline CVD Risk:** All participants have no previous known CVD events (NR) |
| **Study Name:** PRIME study  **Study Location:** Europe (France; Northern Ireland)  **Study Design:** Prospective cohort study  **Follow-up:** 5 yrs and 10 yrs  **Lp(a) Assay:** In-house assay; Immunochemical assay**;** Isoform dependence – NR/unclear  **CVD outcomes:** CHD (fatal or non-fatal MI); angina; CHD (fatal or non-fatal MI and angina) | **Gender:** Males only  **Age:** Elderly 50yrs+ (50 to 59 yrs)  **Ethnicity:** NR/unclear  **Diabetic status:** Mixed population of participants with and without Type 2 diabetes  **Hypertension status:** NR/unclear  **Smoking status:** Mixed smoking status  **Baseline CVD Risk:** All participants have no previous known CVD events (0) |
| **Study Name:** PROCAM  **Study Location:** Europe (Germany)  **Study Design:** Prospective cohort study  **Follow-up:** 8 yrs  **Lp(a) Assay:** Commercial assay; Immunochemical assay**;** Isoform dependence – NR/unclear  **CVD outcomes:** Non-fatal MI, fatal MI, and sudden cardiac death. | **Gender:** Males only  **Age:** Middle aged 40yrs+ (40-65 yrs)  **Ethnicity:** NR/unclear  **Diabetic status:** NR/unclear  **Hypertension status:** NR/unclear  **Smoking status:** NR/unclear  **Baseline CVD Risk:** NR/unclear (NR) |
| **Study Name:** PROSPER  **Study Location:** Europe (Scotland, Ireland, The Netherlands)  **Study Design:** RCT  **Follow-up:** 3.2 yrs (range 2.8 to 4.0)  **Lp(a) Assay:** Commercial assay; Isoform dependence - NR/unclear  **CVD outcomes:** Definite or suspect CHD death, non-fatal MI and fatal or non-fatal stroke; fatal and non-fatal stroke; non-fatal MI. | **Gender:** Mixed male and female  **Age:** Elderly 70yrs+ (70 to 82yrs)  **Ethnicity:** White  **Diabetic status:** Mixed population of participants with and without Type 2 diabetes  **Hypertension status:** Mixture of hypertensive and non-hypertensive patients  **Smoking status:** Current smokers  **Baseline CVD Risk:** Participants had mixed CVD event history (NR) |
| **Study Name:** Quebec Cardiovascular Study  **Study Location:** North America (Canada)  **Study Design:** Prospective cohort study  **Follow-up:** 5ys  **Lp(a) Assay:** Commercial assay; Isoform dependence - NR/unclear  **CVD outcomes:** IHD | **Gender:** Males only  **Age:** Adults ≥ 18 yrs  **Ethnicity:** NR/unclear  **Diabetic status:** Mixed population of participants with and without Type 2 diabetes  **Hypertension status:** NR/unclear  **Smoking status:** Mixed smoking status  **Baseline CVD Risk:** All participants have no previous known CVD events (NR) |
| **Study Name:** RESEARCH  **Study Location:** Europe (The Netherlands)  **Study Design:** Prospective cohort study  **Follow-up:** 6 yrs (median); 8 years (maximum)  **Lp(a) Assay:** Immunochemical assay**;** Isoform dependence - isoform independent  **CVD outcomes:** MACE (death, non-fatal MI, repeat revascularisation) | **Gender:** Mixed male and female  **Age:** Adults ≥ 18 yrs  **Ethnicity:** NR/unclear  **Diabetic status:** Mixed population of participants with and without Type 2 diabetes  **Hypertension status:** Mixture of hypertensive and non-hypertensive patients  **Smoking status:** Mixed smoking status  **Baseline CVD Risk:** All participants have previous known CVD events (History of MI (34%); history of CABG (11%); history of PCI (27%); clinical presentation of stable angine (54%); clinical presentation of unstable angina (28%); clinical presentation of acute MI (18%)) |
| **Study Name:** Reykjavik Study  **Study Location:** Europe (Iceland)  **Study Design:** Nested case control study  **Follow-up:** Mean: 19 yrs  **Lp(a) Assay:** Commercial assay; Immunochemical assay**;** Isoform dependence - NR/unclear  **CVD outcomes:** CHD (non-fatal MI or coronary death) | **Gender:** Mixed male and female  **Age:** Adults ≥ 18 yrs (33-59 yrs)  **Ethnicity:** NR/unclear  **Diabetic status:** Mixed population of participants with and without Type 2 diabetes  **Hypertension status:** Mixture of hypertensive and non-hypertensive patients  **Smoking status:** Mixed smoking status  **Baseline CVD Risk:** All participants have no previous known CVD events (No evidence of CHD or stroke at the baseline examination (ie, participants with electrocardiographic abnormalities or a history of myocardial infarction, angina, or stroke were excluded from the main analyses)) |
| **Study Name:** Rosengren 1990  **Study Location:** Europe (Sweden)  **Study Design:** Case control study  **Follow-up:** 6 yrs  **Lp(a) Assay:** Commercial assay; Immunochemical assay**;** Isoform dependence - NR/unclear  **CVD outcomes:** Deaths from CHD and non-fatal MI | **Gender:** Males only  **Age:** Elderly 50yrs+ (all were aged 50 years at baseline)  **Ethnicity:** NR/unclear  **Diabetic status:** Mixed population of participants with and without Type 2 diabetes  **Hypertension status:** Mixture of hypertensive and non-hypertensive patients  **Smoking status:** Mixed smoking status  **Baseline CVD Risk:** NR/unclear (NR) |
| **Study Name:** Saely 2006  **Study Location:** Europe (Austria)  **Study Design:** Prospective cohort study  **Follow-up:** 3.9±0.8  **Lp(a) Assay:** Commercial assay; Immunoturbidimetric assay**;** Isoform dependence - Isoform independent  **CVD outcomes:** Coronary stenoses (≥ 50%) | **Gender:** Mixed male and female  **Age:** Adults ≥ 18 yrs  **Ethnicity:** White  **Diabetic status:** No participants have Type 2 diabetes  **Hypertension status:** Mixture of hypertensive and non-hypertensive patients  **Smoking status:** Mixed smoking status  **Baseline CVD Risk:** NR/unclear (NR) |
| **Study Name:** Second Northwick Park Heart Study  **Study Location:** Europe (UK)  **Study Design:** Prospective cohort study  **Follow-up:** Mean: 6 years (SD 2yrs)  **Lp(a) Assay:** Commercial assay**;** Isoform dependence - NR/unclear  **CVD outcomes:** Coronary events (sudden cardiac death, acute MI, silent MI, CABG surgery or percutaneous coronary revascularisation). | **Gender:** Males only  **Age:** Elderly 50yrs+  **Ethnicity:** NR/unclear  **Diabetic status:** Mixed population of participants with and without Type 2 diabetes  **Hypertension status:** NR/unclear  **Smoking status:** Current smokers  **Baseline CVD Risk:** All participants have no previous known CVD events (NR) |
| **Study Name:** Strong Health Study  **Study Location:** North America (USA)  **Study Design:** Prospective cohort study  **Follow-up:** NR  **Lp(a) Assay:** Details NR/unclear; Isoform dependence - NR/unclear  **CVD outcomes:** CVD (fatal CVD and non-fatal MI). | **Gender:** Mixed male and female  **Age:** Elderly 50yrs+  **Ethnicity:** NR/unclear  **Diabetic status:** NR/unclear  **Hypertension status:** NR/unclear  **Smoking status:** NR/unclear  **Baseline CVD Risk:** All participants have no previous known CVD events (134 fatal and 254 nonfatal CVD cases) |
| **Study Name:** SHDC, WHO MONICA and VIP  **Study Location:** Europe (Sweden)  **Study Design:** Nested case control  **Follow-up:** NR  **Lp(a) Assay:** In-house; immunochemical assay**;** Isoform dependence - NR/unclear  **CVD outcomes:** MI | **Gender:** Male  **Age:** Adults ≥ 18yrs  **Ethnicity:** NR/unclear  **Diabetic status:** Mixed population of participants with and without Type 2 diabetes  **Hypertension status:** Mixture of hypertensive and non-hypertensive patients  **Smoking status:** Mixed smoking status  **Baseline CVD Risk:** Participants had mixed CVD event history (mixture of those who have (n=62) - cases, and those who haven't (n=124) had MI – controls) |
| **Study Name:** TNT Study  **Study Location:** Worldwide (Australia; USA; Austria; Belgium; Canada; France; Germany; Ireland; Italy; Netherlands; South Africa; Spain; Switzerland; UK)  **Study Design:** RCT  **Follow-up:** 4.9 yr  **Lp(a) Assay:** Commercial assay; Immunoturbidimetric assay; Isoform dependence - NR/unclear  **CVD outcomes:** CHD death; non-fatal, non-procedure-related myocardial infarction; resuscitated cardiac arrest; and fatal or nonfatal stroke. | **Gender:** Mixed male and female  **Age:** Middle aged 40yrs+ (35-75yrs)  **Ethnicity:** NR/unclear  **Diabetic status:** Mixed population of participants with and without Type 2 diabetes  **Hypertension status:** Mixture of hypertensive and non-hypertensive patients  **Smoking status:** Mixed smoking status  **Baseline CVD Risk:** All participants have previous known CVD events |
| **Study Name:** Tromso Study  **Study Location:** Europe (Norway)  **Study Design:** Nested case control study  **Follow-up:** 10yrs  **Lp(a) Assay:** Commercial assay; Immunoassay**;** Isoform independent  **CVD outcomes:** MI | **Gender:** Mixed male and female  **Age:** Elderly 60yrs+ (approximately 60 yrs)  **Ethnicity:** Predominantly White  **Diabetic status:** NR/unclear  **Hypertension status:** Mixture of hypertensive and non-hypertensive patients  **Smoking status:** Mixed smoking status  **Baseline CVD Risk:** Cases were defined as all participants with no previous MI, ischemic stroke, CABG, percutaneous coronary intervention, or self-reported angina at baseline and who experienced a first-ever MI (n=419) within 10yrs of follow-up. Controls (n=398) were randomly selected from the entire group of participants completing 10yr follow-up without an event of interest and using the same inclusion criteria as for the cases. |
| **Study Name:** ULSAM  **Study Location:** Europe (Sweden)  **Study Design:** Prospective cohort study  **Follow-up:** Median follow-up time (range): 29.3 (0.04 to 32.7)  **Lp(a) Assay:** Commercial assay; Radioimmunoassay**;** Isoform dependence - NR/unclear  **CVD outcomes:** Fatal or nonfatal stroke or TIA (ICD-9 codes 430 to 32 and 434 to 36, ICD-10 codes I60-I64, I66 and G45) | **Gender:** Males only  **Age:** Elderly 50yrs+ (approximately 50 yrs)  **Ethnicity:** NR/unclear  **Diabetic status:** NR/unclear  **Hypertension status:** Mixture of hypertensive and non-hypertensive patients  **Smoking status:** Mixed smoking status  **Baseline CVD Risk:** Participants had mixed CVD event history (Left Ventricular Hypertrophy shown in ECG (ECG-LVH) 1.4%) |
| **Study Name:** Wehinger 1999  **Study Location:** Europe (Germany)  **Study Design:** Prospective cohort study  **Follow-up:** 1 yr  **Lp(a) Assay:** Commercial assay; Immunonephelometric assay**;** Isoform dependence - NR/unclear  **CVD outcomes:** Major adverse cardiac events such as cardiac- or procedure-related death, MI and target lesion revascularization | **Gender:** Mixed male and female  **Age:** Adults ≥ 18 yrs  **Ethnicity:** NR/unclear  **Diabetic status:** Mixed population of participants with and without Type 2 diabetes  **Hypertension status:** Mixture of hypertensive and non-hypertensive patients  **Smoking status:** Mixed smoking status  **Baseline CVD Risk:** All participants have previous known CVD events (All patients had symptomatic CAD) |
| **Study Name:** WHI-OS/HaBPS  **Study Location:** North America (USA)  **Study Design:** Nested case control study  **Follow-up:** Mean: 7.9yrs (range 1.9 to 10.5yrs)  **Lp(a) Assay:** Details NR/unclear; Isoform dependence - NR/unclear  **CVD outcomes:** Ischemic stroke | **Gender:** Females only  **Age:** Elderly 50yrs+ (50-79yrs)  **Ethnicity:** Mixed  **Diabetic status:** Mixed population of participants with and without Type 2 diabetes  **Hypertension status:** NR/unclear  **Smoking status:** Mixed smoking status  **Baseline CVD Risk:** All participants have no previous known CVD events (NR) |
| **Study Name:** WHS  **Study Location:** North America (USA)  **Study Design:** RCT  **Follow-up:** Median: 12.3yrs  **Lp(a) Assay:** Commercial assay; Immunoturbidimetric assay**;** Isoform dependence - isoform independent  **CVD outcomes:** PAD (intermittent claudication or peripheral artery surgery); TIA | **Gender:** Females only  **Age:** Elderly 50yrs+ (≥ 45yrs)  **Ethnicity:** White  **Diabetic status:** Mixed population of participants with and without Type 2 diabetes  **Hypertension status:** Mixture of hypertensive and non-hypertensive patients  **Smoking status:** Mixed smoking status  **Baseline CVD Risk:** All participants have no previous known CVD events (NR) |
| **Study Name:** Zimmermann 1999  **Study Location:** Europe (Germany)  **Study Design:** Prospective cohort study  **Follow-up:** At 12 and 24 mths after baseline examination  **Lp(a) Assay:** Details NR/unclear; Isoform dependence - NR/unclear  **CVD outcomes:** Death, CVD death, stroke, HF, MI | **Gender:** Mixed male and female  **Age:** Adults ≥ 18 yrs  **Ethnicity:** White  **Diabetic status:** Mixed population of participants with and without Type 2 diabetes  **Hypertension status:** NR/unclear  **Smoking status: Mixed smoking status**  **Baseline CVD Risk:**  Participants had mixed CVD event history |

CAD coronary artery disease; CV cardiovascular; CVD cardiovascular disease; CHD coronary heart disease; ECG electrocardiogram; HF heart failure; ICD International Classification of Diseases; IHD ischemic heart disease; LVH left ventricular hypertrophy; MACE major adverse cardiac events; MI myocardial infarction; mth months; NR not reported; PAD peripheral arterial disease; RCT randomised controlled trial; TIA transient ischemic attack; UK United Kingdom; USA United States of America; WHO World Health Organisation; yrs years
