# Supplementary material for: The relationship between Lp(a) and CVD outcomes: a systematic review
Source: Lipids Health Dis. 2016 May 17;15:95. doi: 10.1186/s12944-016-0258-8 (PMC4869344; doi:10.1186/s12944-016-0258-8)
Supplement: Additional file 3: Table S2. — Summary of QUIPS quality assessment domains (60 studies). (DOCX 54 kb) [file 12944_2016_258_MOESM3_ESM.docx]

**Additional file 3**

**Table S2: Summary of QUIPS quality assessment domains (60 studies)**

| **Study ID** | **Domain 1: STUDY PARTICIPANTS** | **Domain 2: STUDY ATTRITION** | **Domain 3: PROGNOSTIC FACTOR MEASUREMENT** | **Domain 4: OUTCOME MEASUREMENT** | **Domain 5: STUDY CONFOUNDING** | **Domain 6: STATISTICAL ANALYSIS AND REPORTING** | **OVERALL RISK OF BIAS** |
| --- | --- | --- | --- | --- | --- | --- | --- |
| 4S Study | Moderate | Low | Moderate | Low | Low | Moderate | Moderate |
| Agewall 2002 | High | High | High | Low | Low | Moderate | High |
| AIM–HIGH | Low | Low | Low | Low | Low | Low | Low |
| ARIC | Low | Not enough information | Not enough information | Moderate | Moderate | Not enough information | Not enough information |
| Bruneck | Not enough information | Not enough information | Moderate | Moderate | Low | Low | Not enough information |
| BUPA Study | High | Not enough information | High | Low | Low | Moderate | High |
| Caerphilly Study | Low | Low | Low | Low | Low | Low | Low |
| CCHS | Moderate | Low | Moderate | Low | Low | Moderate | Moderate |
| CGPS/CCHS | Not enough information | Not enough information | High | Not enough information | Moderate | Low | High |
| Chin-Shan Community Cardiovascular Cohort Study | Low | Moderate | Low | Low | Moderate | Low | Moderate |
| Cho 2010 | Low | High | Moderate | Moderate | Moderate | Low | High |
| CHOICE | Low | Moderate | Low | Low | Low | Low | Moderate |
| CHS | Moderate | Not enough information | Low | Low | Low | Low | Not enough information |
| Cleveland Clinic Hemodialysis Cohort | Moderate | Low | Moderate | Low | Low | Low | Moderate |
| D'Angelo 2006 | Low | Not enough information | Low | Low | Low | Low | Not enough information |
| Diamant Alpin Collaborative Dialysis Cohort | Low | Not enough information | High | Low | Moderate | Low | High |
| Edinburgh Artery Study | Moderate | Low | Moderate | Low | Low | Low | Moderate |
| EPIC | Low | Low | Moderate | Low | Moderate | Low | Moderate |
| Ezhov 2014 | Low | Low | Low | Low | Low | Low | Low |
| FHS | Low | Moderate | Low | Low | Low | Low | Moderate |
| FINRISK '92 Hemostasis Study | Low | Not enough information | Moderate | Low | Not enough information | Low | Not enough information |
| Fletcher Challenge study | Low | Low | Moderate | Low | Low | Low | Moderate |
| GENERATION | Low | Moderate | Not enough information | Low | Low | Low | Not enough information |
| GRIPS | Not enough information | Moderate | Low | Moderate | Low | Low | Not enough information |
| HERS | Low | Not enough information | Low | Low | Low | Low | Not enough information |
| HHS | Low | Moderate | Moderate | Low | Low | Low | Moderate |
| HPFS | Moderate | Moderate | Moderate | Moderate | Low | Low | Moderate |
| HPFS and NHS Study | Moderate | Low | Moderate | Low | Low | Low | Moderate |
| Ikenaga 2011 | Moderate | Not enough information | Not enough information | Not enough information | Not enough information | Moderate | Not enough information |
| ILSA | Moderate | Moderate | Moderate | Moderate | Moderate | High | High |
| InCHIANTI Study | High | High | High | Moderate | Low | Low | High |
| JDCS | Low | High | High | Low | Moderate | Low | High |
| JUPITER | Low | Moderate | Moderate | Low | Moderate | Low | Moderate |
| Kangbuk Samsung Health study* | Not enough information | Not enough information | Not enough information | Not enough information | Not enough information | Not enough information | Not enough information |
| Koda 1999 | Low | High | Moderate | Moderate | Not enough information | Not enough information | High |
| Konishi 2013 | Low | High | High | Moderate | Low | Low | High |
| Kwon 2015 | Low | Moderate | Low | Low | Low | Low | Moderate |
| LIPID | Low | Moderate | Moderate | Moderate | Moderate | Low | Moderate |
| Lipid Research Clinics Coronary Primary Prevention Trial | Low | Moderate | High | Low | Moderate | Low | High |
| Luc 2002 | Moderate | Moderate | Moderate | Moderate | Low | Low | Moderate |
| MEGA Study | Moderate | Low | Moderate | Low | Low | Low | Moderate |
| Park 2015 | Low | Low | Not enough information | Moderate | Low | Low | Moderate |
| PHS | Moderate | Low | Moderate | Low | Low | Moderate | Moderate |
| PROCAM | Not enough information | Moderate | Moderate | Moderate | High | Moderate | High |
| PROSPER | Low | Moderate | Low | Low | Low | Low | Moderate |
| Quebec Cardiovascular Study | Moderate | Moderate | Moderate | Low | Moderate | Low | Moderate |
| RESEARCH | Low | Moderate | Moderate | Moderate | Moderate | Low | Moderate |
| Reykjavik Study | Low | Low | Moderate | Moderate | Moderate | Low | Moderate |
| Rosengren 1990 | Low | Moderate | Moderate | Low | Moderate | Low | Moderate |
| Saely 2006 | Low | Moderate | Low | low | low | low | Moderate |
| Second Northwick Park Heart Study | Low | Low | Low | Moderate | Moderate | Moderate | Moderate |
| Strong Health Study | Moderate | Low | Low | Moderate | Moderate | Moderate | Moderate |
| SHDC, WHO MONICA and VIP | Low | Low | Not enough information | Low | Moderate | Low | Not enough information |
| TNT Study | High | Low | Not enough information | Low | Moderate | Low | High |
| Tromoso Study | Low | Low | Moderate | Low | Moderate | Low | Moderate |
| ULSAM | Low | Low | Low | Low | low | Low | Low |
| Wehinger 1999 | Low | Low | Moderate | Low | Moderate | Low | Moderate |
| WHI-OS/HaBPS | Low | Moderate | Moderate | Low | Low | Low | Moderate |
| WHS | Moderate | Moderate | Moderate | Low | Moderate | Moderate | Moderate |
| Zimmermann 1999 | Low | Low | Low | Low | low | Low | Low |

* Assessment based only on abstract publication
